# Novel polycyclic “turn-on” and “turn-off” pyrazoline and pyrazole fluorescent sensors for selective real-world monitoring of Fe^3+^/Fe^2+^ in aqueous environments†

**DOI:** 10.1039/d4ra06457g

**Published:** 2024-10-31

**Authors:** Alexander Ciupa

**Affiliations:** a Materials Innovation Factory, University of Liverpool 51 Oxford Street Liverpool L7 3NY UK ciupa@liverpool.ac.uk

## Abstract

Seven novel polycyclic pyrazoline and pyrazole sensors were synthesised and screened for useful photophysical properties with pyrazoline 2 and pyrazole 7, displaying an Fe^3+^ “turn-off” response in aqueous environments with Fe^3+^ limits of detection (LoD) of 2.12 μM and 3.41 μM, respectively. Both 2 and 7 sensors functioned in aqueous environments with real-world examples of Fe^3+^ detection in tap water and mineral water samples. 2 and 7 are suitable for the detection of Fe^3+^ at concentrations below the maximum iron limits for drinking water set by the Environmental Protection Agency (EPA) and European Union (EU).

## Abstract 2

Seven novel polycyclic pyrazoline and pyrazole sensors were synthesised and screened for useful photophysical properties with pyrazoline 2 and pyrazole 7, displaying an Fe^3+^ “turn-off” response in aqueous environments.

## Graphical abstract

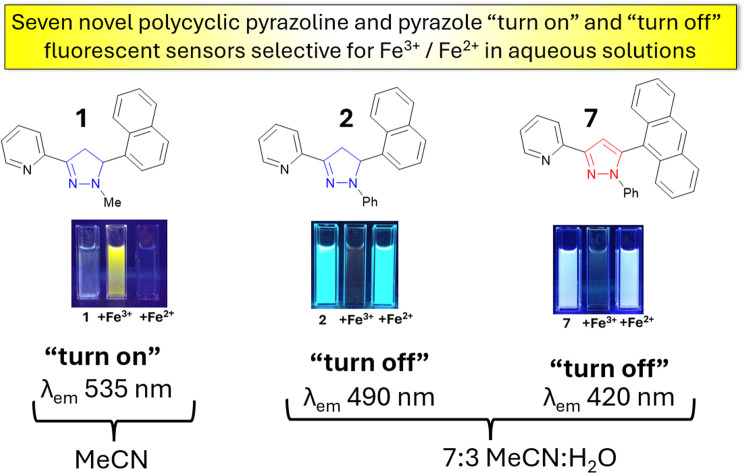

## Introduction

**Scheme 1:**
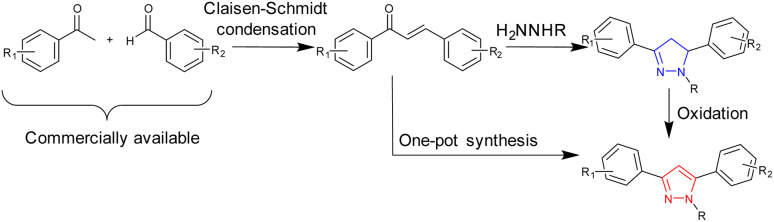
Synthesis of pyrazoline and pyrazoles from chalcone precursors.

**Fig. 1:**
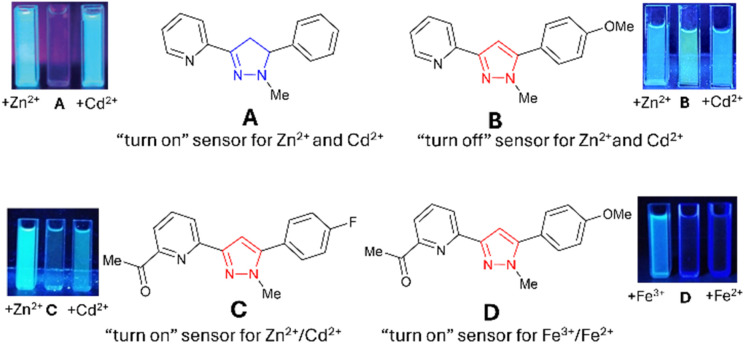
Recently reported pyrazoline and pyrazole fluorescent sensors; images are reproduced from ref. 15–17.

Iron is the most abundant transition metal in the human body^1^ and is vital for a range of biological functions, including oxygen transport *via* haemoglobin,^2^ catalytic activity of iron oxygenases,^3^ and DNA synthesis and repair.^4^ Ferric (Fe^3+^) iron and ferrous (Fe^2+^) iron are the two predominant forms of iron in the human body with the redox cycling between oxidation states being pivotal to their biological functions.^1,5^ Excess iron is linked to numerous medical problems,^6^ including hemochromatosis,^7^ Alzheimer's disease, and Parkinson's disease.^8,9^ Therefore, regular monitoring of iron intake is of paramount importance. The Environmental Protection Agency (EPA) in the USA has set the iron limit in drinking water at 5.4 μM,^10^ whereas the European Union (EU) has set it at 3.5 μM.^11^ Fluorescence spectroscopy offers many advantages in monitoring iron levels in drinking water, including a low limit of detection, high specificity and the ability to fine-tune the fluorescence emission wavelength (*λ*_em_).^12,13^ Pyrazoline,^14^ a five-membered heterocycle with two adjacent nitrogen atoms (blue in Scheme 1 and Fig. 1), has well-established fluorescent properties with sensors reported for Zn^2+^,^18^ Al^3+^,^19^ and Fe^3+^.^20^ Pyrazole^21^ (red in Scheme 1 and Fig. 1) is closely related to pyrazolines and displays useful fluorescent properties.^16,17^ Chalcones^22,23^ are versatile precursors enabling the generation of large libraries of pyrazolines and pyrazoles *via* short (2–3 step) syntheses (Scheme 1) from commercially available starting materials.

“Turn-on” sensors display an increased *λ*_em_ in the presence of an analyte, for example, A with Zn^2+^ and Cd^2+^ (Fig. 1).^15^ “Turn-off” sensors display reductions in the *λ*_em_ with an analyte; for example, B demonstrates a minor reduction in the *λ*_em_ with Zn^2+^ and Cd^2+^. Recent studies highlighted that the addition of an acetyl side group adjacent to the chelation site greatly enhanced analyte selectivity, for example, Zn^2+^/Cd^2+^ for C and Fe^3+^/Fe^2+^ for D.^16,17^ A variety of mechanistic pathways can account for an increased *λ*_em_, including blocking of photoinduced electron transfer (PET)^24^ and the chelation enhanced fluorescence effect (CHEF).^25^ A decreased *λ*_em_ can result from the chelation enhancement quenching effect (CHEQ)^26^ or fluorescence resonance energy transfer (FRET).^27^

While sensors A–D confirm that structural complexity is not a prerequisite for complex functionality, their potential as fluorescent sensors to date has been limited to organic solvents.^15–17^ A key requirement for real-world monitoring is a fluorescent response in aqueous solutions. To address this research need and further investigate how to fine-tune the photophysical properties of these sensors, we developed seven novel sensors incorporating phenyl, naphthalene and anthracene units. Incorporation of one or more polycyclic component into sensor design has been shown to confer favourable fluorescent properties.^18*a*,28^ The two lead sensors (2 and 7) were analysed in aqueous solution, confirming that pyrazoline A and pyrazole B can transition from purely organic solvent sensors to sensors that are functional in mixed organic and aqueous solutions with minor modifications. The lead sensors displayed excellent selectivity for Fe^3+^/Fe^2+^, with the Fe^3+^ limit of detections below the USA and EU iron drinking water limit, validating their application in real-world monitoring of iron concentrations in drinking water.

## Results and discussion

**Scheme 2:**
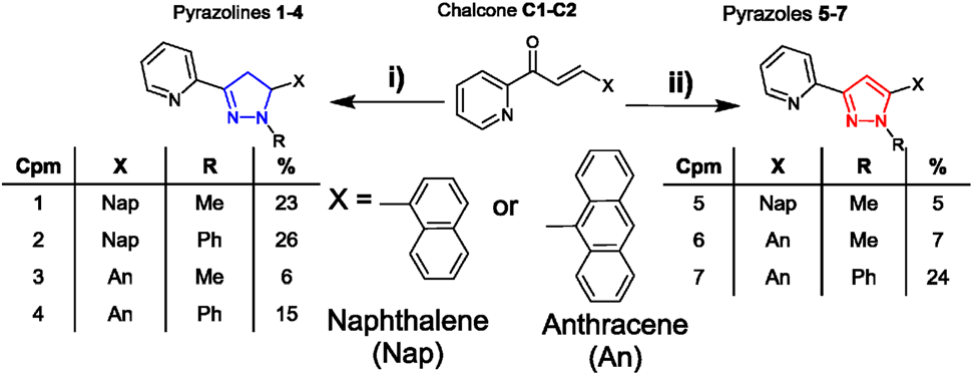
Synthesis of seven novel sensors from shared chalcone precursors: (i) 2.0 equivalent hydrazine (H_2_NNHMe or H_2_NNHPhe), MeOH, 60 °C, and 24 h (ref. 15) (ii) is the same as (i) + 1.0 equivalent CuCl_2_.^16^

Chalcone precursors C1 with naphthalene (X = Nap) and C2 with anthracene (X = An) units were prepared (ESI S1†) *via* literature methods^29,30^ in excellent yield (75–93%) (Scheme 2). The pyrazoline series 1–4 was synthesised by adapting previously used methods^15,17^ in acceptable yields. The pyrazole series 5–7 were synthesised using a direct chalcone-to-pyrazole one-pot method.^16^ With seven novel sensors in hand, we investigated their photophysical properties initially in MeCN. MeCN was selected to allow direct comparison with three previous studies on the structurally related sensors A–D, which was also performed in MeCN. The lead sensors were then investigated in a mixed organic aqueous solution of 7 : 3 MeCN : H_2_O. Well established protocols for screening fluorescent sensors in organic solvents were followed throughout the study.^28^

**Fig. 2:**
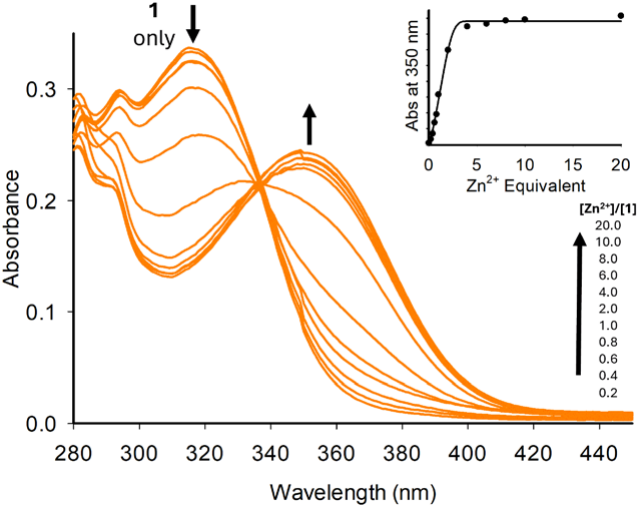
UV/vis study for 1 (20 μM, MeCN) with 0–20 equivalents of Zn^2+^.

UV/vis spectroscopy was used to confirm that 1–7 undergo chelation. We initially used the group 12 metals (Zn^2+^, Cd^2+^ and Hg^2+^), as structurally similar sensors A and B are known to chelate this metal group (Fig. 2 for 1 + Zn^2+^, and ESI S4† for 2–7). Upon increasing equivalents of Zn^2+^, the initial absorbance band at 315 nm decreased with the formation of a new absorbance band at 350 nm up to 5.0 equivalents of Zn^2+^ (Fig. 2). Further additions resulted in a plateau at 350 nm. A similar trend was observed with the pyrazole series (see ESI S4† for 2–7).

**Fig. 3:**
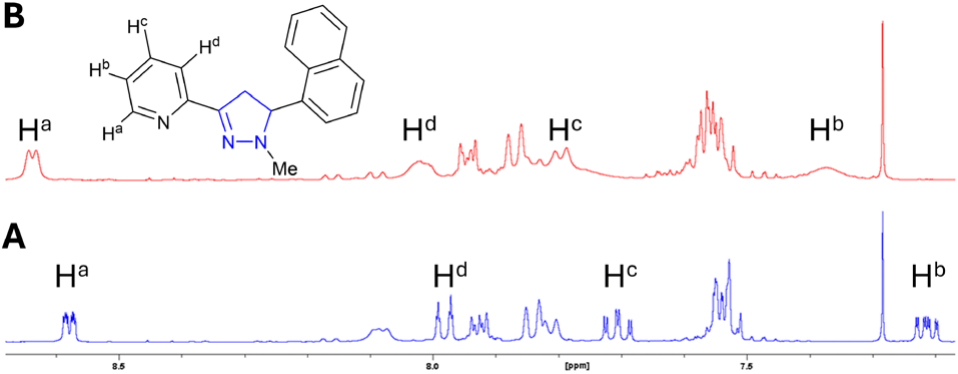
Partial ^1^H NMR spectra: (A) is 1 (20 μM, CDCl_3_) and (B) is 1 + 2.0 equivalents of Zn^2+^.

^1^H NMR studies with and without Zn^2+^ were performed to confirm that 1–4 underwent chelation, with the results from 1 representative of all pyrazolines (Fig. 3 for 1, ESI S3† for 2–4). Upon addition of 2.0 equivalents of Zn^2+^, the pyridine protons demonstrated broadening and downfield movement in the chemical shift; for example, H^a^ from 8.57 ppm to 8.63 ppm, H^d^ from 7.97 ppm to 8.02 ppm, and H^c^ from 7.70 ppm to 7.90 ppm (Fig. 3A and B). This is characteristic of chelation, and has been reported previously for A (ref. 15) and other sensors.^20,28*a*,*e*,31*a*,*d*,*e*^

**Fig. 4:**
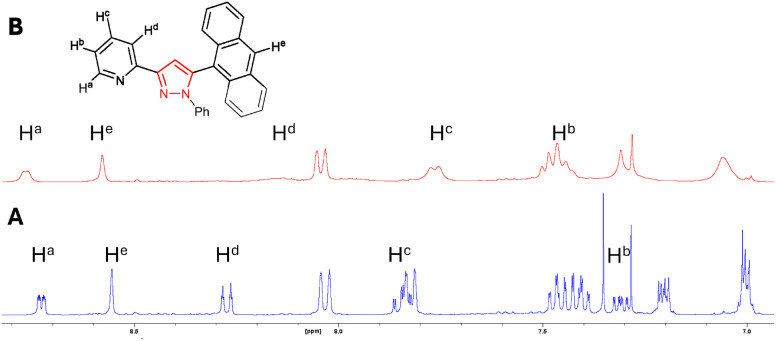
Partial ^1^H NMR spectra: (A) is 7 (15 μM, CDCl_3_) and (B) is 7 + 2.0 equivalents Zn^2+^.

A further ^1^H NMR study was conducted for pyrazole 7 with a similar trend upon the addition of 2.0 equivalents of Zn^2+^ (Fig. 4). The chemical shift of pyridine protons H^a^ increased from 8.73 ppm to 8.77 ppm, while that of the anthracene singlet H^e^ increased from 8.55 ppm to 8.68 ppm and pyridine H^b^ from 7.31 ppm to 7.45 ppm. Broadening of all signals was also observed upon the addition of Zn^2+^, confirming chelation and agrees with a similar study conducted on C.^17^ These results indicate that the chelate site is centred about the pyridine ring, and chelation occurs with either a pyrazoline or pyrazole heterocycle, or a naphthalene or anthracene side unit.

**Fig. 5:**
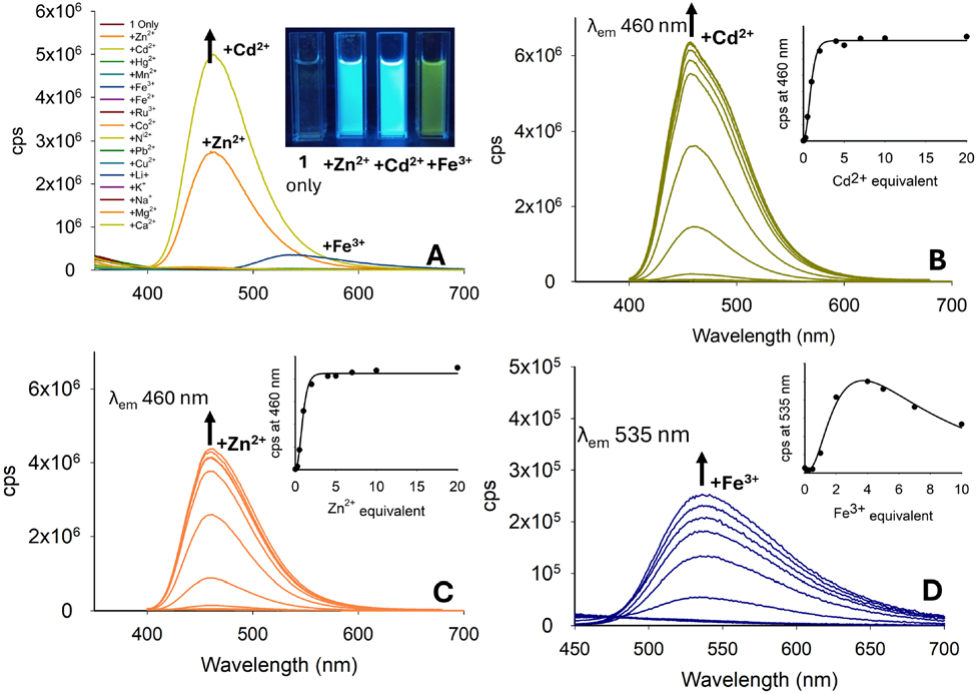
Photophysical properties of 1 (20 μM, MeCN). (Panel A) Metal screen at *λ*_ex_ 280 nm with 5.0 equivalents of the indicated metal. (Panels B–D) Titration studies for Cd^2+^, Zn^2+^ and Fe^3+^, respectively; cps is counts per second. Cuvette images were taken under the irradiation of a 100 W *λ*_ex_ 365 nm lamp.

The fluorescence response of pyrazoline 1 with a range of metals was investigated in MeCN with increased *λ*_em_ 460 nm with Cd^2+^ and Zn^2+^ (Fig. 5A), analogous to that of pyrazoline A, which was previously reported. A linear response of up to 5.0 equivalents of both Cd^2+^ and Zn^2+^, reaching a plateau on further addition, was observed (Fig. 5B and C). An unexpected “turn-on” response for Fe^3+^ at *λ*_em_ 535 nm suggested that 1 could function as a multi-analyte sensor, as observed in previous studies.^32^ This response was not observed in sensor A, suggesting that the naphthalene unit was responsible for this additional feature. The increase at *λ*_em_ 535 nm upon addition of Fe^3+^ peaked at 5.0 equivalents of Fe^3+^, with further addition reducing the fluorescence, which may be possibly due to paramagnetic quenching of the excited state (Fig. 5D).

**Fig. 6:**
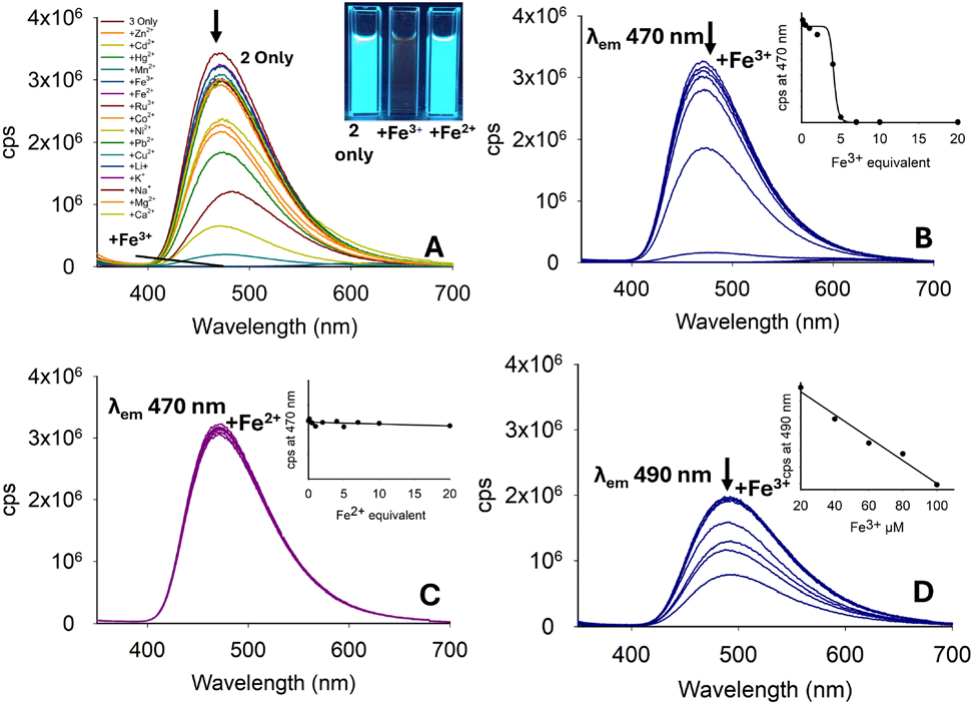
Photophysical properties of 2 (20 μM, MeCN). (Panel A) is the metal screen at *λ*_ex_ 280 nm with 5.0 equivalents of the indicated metal. (Panels B and C) are titration studies for Fe^3+^ and Fe^2+^ in MeCN, respectively. (Panel D) is the Fe^3+^ titration in 7 : 3 MeCN : H_2_O; cps is counts per second. Cuvette images were taken under the irradiation of a 100 W *λ*_ex_ 365 nm lamp.

Repeating the Cd^2+^ titration study for pyrazoline 1 in a 7 : 3 MeCN : H_2_O solution resulted in an approx. 90% reduction in the “turn-on” response (see ESI S5†), greatly hindering the potential of 1 in aqueous environments. Previous sensors A–D and pyrazoline 1 all had a methyl substituent on the nitrogen ring. In pyrazoline 2, this was replaced by a phenyl unit and unexpectedly reversed the fluorescence response from “turn-on” to “turn-off”, which was most noticeable for Fe^3+^ (Fig. 6A). The *λ*_em_ at 470 nm for 2 only (*φ*_f_ 0.74) decreased with up to 5.0 equivalents Fe^3+^ (*φ*_f_ < 0.01), and further addition resulted in complete quenching of the fluorescence (Fig. 6B). A Fe^2+^ titration study demonstrated no significant change in *λ*_em_ 470 nm with up to 20.0 equivalents (Fig. 5C), suggesting that pyrazoline 2 could selectively detect iron in the +3 oxidation state over iron in the +2 oxidation state (Fe^3+^/Fe^2+^). A Fe^3+^ titration study in 7 : 3 MeCN : H_2_O was conducted to determine if this “turn-off” response remained in aqueous samples (see Fig. 6D for Fe^3+^, and ESI S5† for the full metal screen). The presence of water reduced *λ*_em_ by approximately 30% (*φ*_f_ 0.83), but retained a linear (*R*^2^ = 0.984) reduction in *λ*_em_ (*φ*_f_ 0.07) in the concentration range between 20–100 μM Fe^3+^, suggesting that pyrazoline 2 could be utilised for the selective quantification of Fe^3+^ in aqueous samples (Fig. 6D). A slight increase of 20 nm in *λ*_em_ from 470 nm in 100% MeCN to 490 nm in 7 : 3 MeCN : H_2_O was observed (Fig. 5D).

**Fig. 7:**
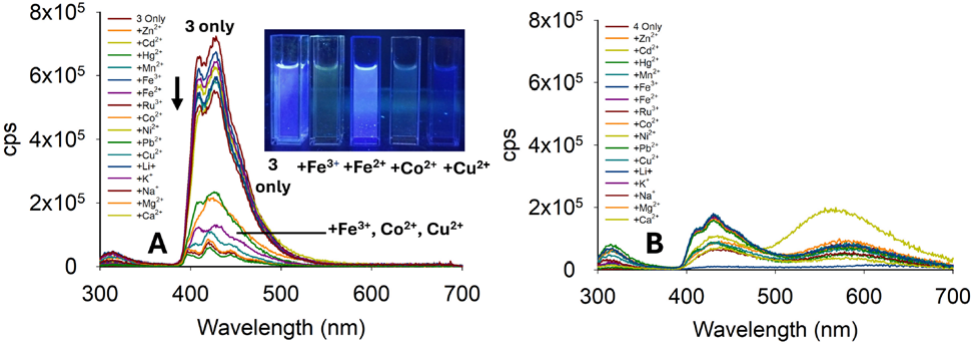
Photophysical properties of 3 (20 μM, MeCN) (Panel A) and 4 (Panel B) metal screen at *λ*_ex_ 250 nm with 5.0 equivalents of the indicated metal; cps is counts per second. Cuvette images were taken under the irradiation of a 100 W *λ*_ex_ 365 nm lamp.

Our focus shifted to the anthracene pyrazolines 3–4 to determine if a third aromatic ring would confer beneficial properties. Pyrazoline 3 displayed a “turn-off” response for Fe^3+^/Fe^2+^ at 420 nm. However, several other metals produced a “turn-off” response that was equal to or greater than that of Fe^3+^; for example, Co^2+^ and Cu^2+^ (Fig. 7A). The *λ*_em_ of 3 only was significantly less than that of 2, further limiting the application of this pyrazoline as a sensor.

**Fig. 8:**
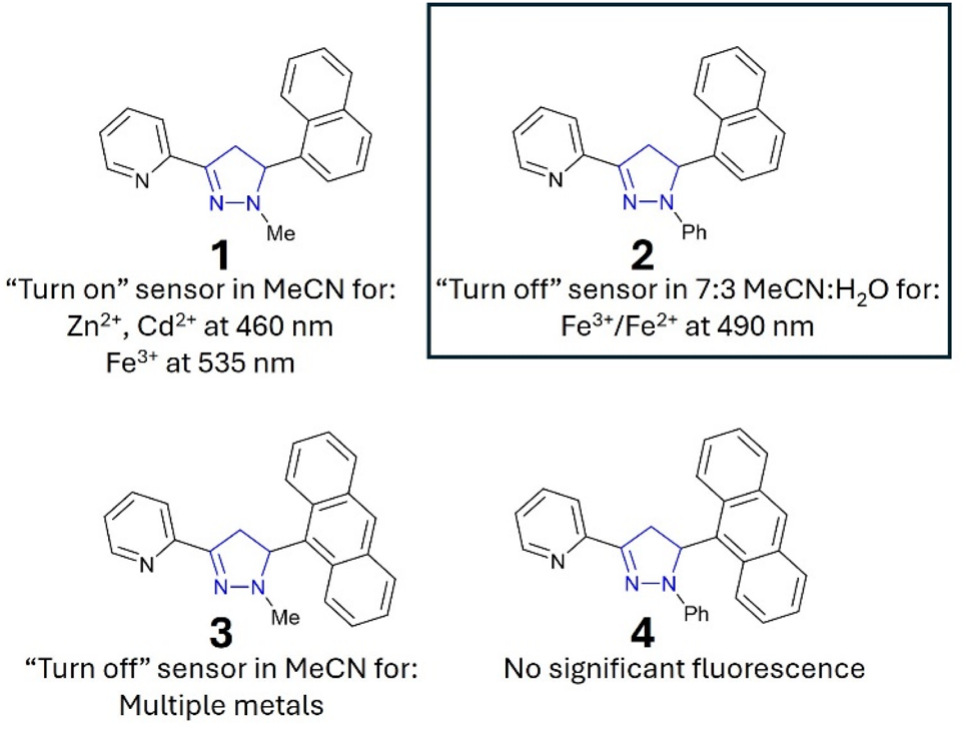
Summary of the pyrazoline series. 2 (inset) was selected for further investigation.

Pyrazoline 4 contained a phenyl instead of a methyl group. This change significantly altered the photophysical properties of 1 and 2. Pyrazoline 4 was also analysed, and displayed very weak fluorescence at 420 nm with insignificant differences observed on addition of a range of metals (Fig. 7B). The *λ*_em_ of 4 was approximately a third of the value for 3, suggesting that 4 was also unsuitable for sensing. In summary, the two anthracene pyrazolines did not display improved fluorescence properties over the naphthalene sensors. A summary of the pyrazoline series is displayed in Fig. 8.

**Fig. 9:**
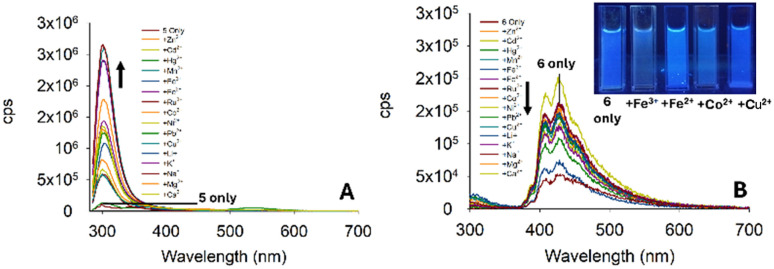
(Panel A) Metal screen for 5 (20 μM, MeCN) at *λ*_ex_ 280 nm with 5.0 equivalents of the indicated metal. (Panel B) Metal screen for 6 at *λ*_ex_ 250 nm in MeCN; cps is counts per second. Cuvette images were taken under the irradiation of a 100 W *λ*_ex_ 365 nm lamp.

With the initial studies on the pyrazoline series complete and pyrazoline 2 selected for further investigation, we performed a similar analysis on the pyrazole series 5–7. To our surprise, pyrazole 5 displayed a “turn-on” response at 310 nm (Fig. 9A), whereas pyrazole 6 with an additional aryl ring displayed a “turn-off” response to a variety of metals (Fig. 7B). Unfortunately, no selectively to Fe^3+^ was observed. Therefore, these pyrazoles were not selected for further investigation.

**Fig. 10:**
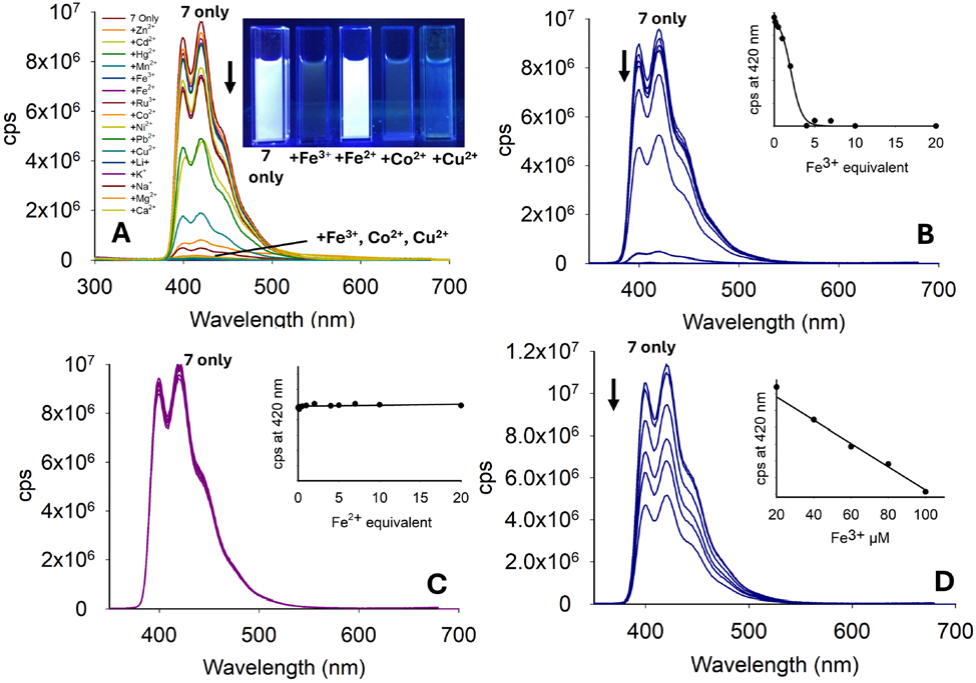
Photophysical properties of 7 (20 μM, MeCN). (Panel A) Metal screen at *λ*_ex_ 280 nm with 5.0 equivalents of the indicated metal. (Panels B and C) Titration studies for Fe^3+^ and Fe^2+^ in MeCN, respectively. (Panel D) Fe^3+^ titration in 7 : 3 MeCN : H_2_O; cps is counts per second. Cuvette images were taken under the irradiation of a 100 W *λ*_ex_ 365 nm lamp.

**Fig. 11:**
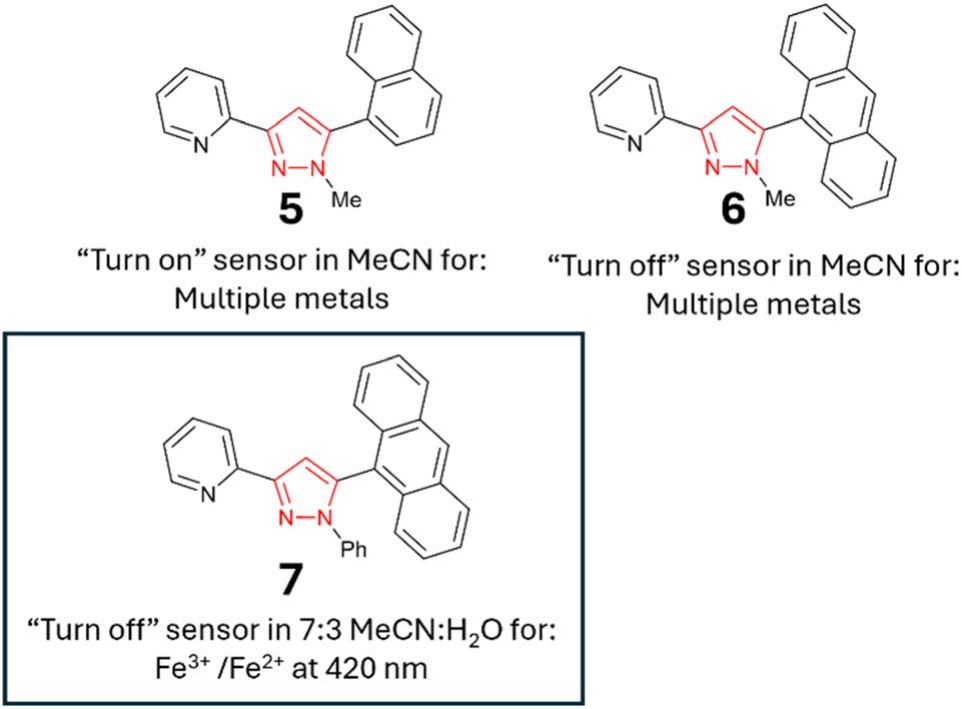
Summary of the pyrazole series; 7 (inset) was selected for further investigation.

Pyrazole 7 with a phenyl unit on the pyrazole nitrogen displayed significant *λ*_em_ intensity at 420 nm in the absence of metals (*φ*_f_ 0.33), and this was diminished only in the presence of a small number of metals, most noticeably Fe^3+^, Co^2+^ and Cu^2+^ (Fig. 10A). Significant reduction in *λ*_em_ at 420 nm was observed upon the addition of 4.0 equivalents of Fe^3+^ (*φ*_f_ < 0.01). Further addition of Fe^3+^ resulted in complete quenching of the fluorescence intensity (Fig. 10B). A similar study with Fe^2+^ demonstrated no reduction, showing excellent Fe^3+^/Fe^2+^ selectivity (Fig. 10C). Repeating the Fe^3+^ titration in a 7 : 3 MeCN : H_2_O solution demonstrated an excellent “turn-off” response with a linear reduction in *λ*_em_ (*R*^2^ = 0.978) (*φ*_f_ 0.43 to *φ*_f_ 0.18), suggesting this would be a suitable sensor for Fe^3+^ in aqueous environments (see Fig. 10D for Fe^3+^, and ESI† for the full metal screen). A summary of the pyrazole series is displayed in Fig. 11 and pyrazole 7 was selected for further investigation.

**Fig. 12:**
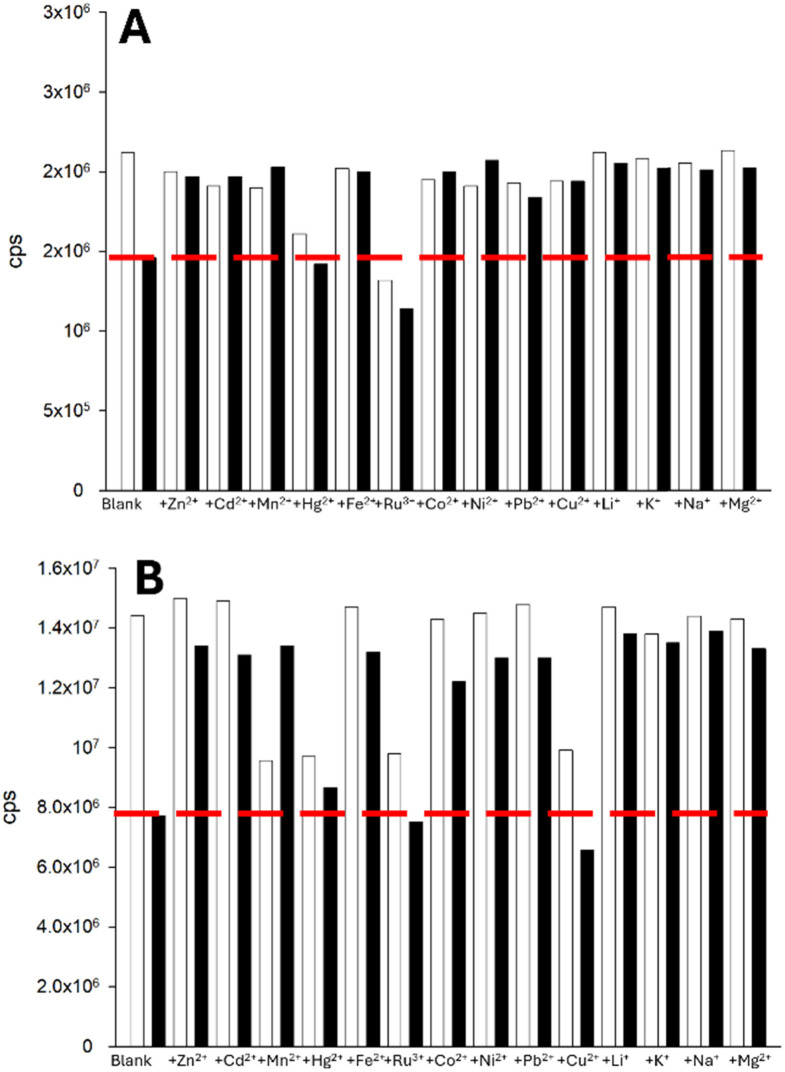
Competition assays for 2 (Panel A) and 7 (Panel B). The white bar represents the sensor (20 μM, MeCN) with 5.0 equivalents of the indicated cation; the black bars is the same with 5.0 equivalents Fe^3+^ after equilibrating for 3 min. 2*λ*_ex_ 280 nm with *λ*_em_ 490 nm; 7*λ*_ex_ 250 nm with *λ*_em_ 420 nm.

With two lead sensors selected, one pyrazoline and one pyrazole, we performed a range of competition assays to determine if the Fe^3+^-triggered fluorescence “turn-off” response is retained in the presence of competing cations. Pyrazoline 2 was the most affected by competition across the range of cations screened, except for Ru^3+^ (Fig. 12A). Pyrazole 7 was similarly impacted by the presence of competing cations, except for Ru^3+^ and Cu^2+^ (Fig. 12B). This result suggests that neither naphthalene or anthracene, nor pyrazoline or pyrazole, are responsible for the high competition observed. It is highly possible that the large open chelation site, as demonstrated from a previous X-ray crystal structure for the pyrazole analogue of A,^15^ is responsible. Restricting the chelation site (for example, by the addition of an acetyl group) should be further explored in future analogues of 2 and 7, as this was shown to be highly effective for C and D.^17^

**Fig. 13:**
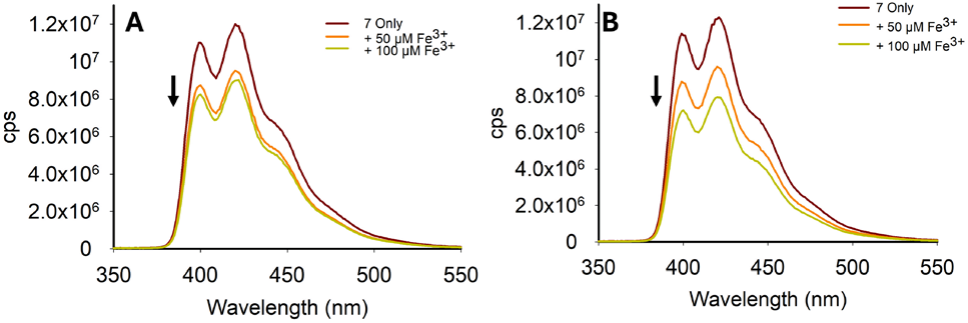
Fe^3+^-triggered “turn-off” response for 7 in tap water (Panel A). Fe^3+^-triggered “turn-off” response for 7 in mineral water (Panel B), *λ*_ex_ 250 nm. Solvent was 7 : 3 MeCN : H_2_O; cps is counts per second.

A study was conducted to determine the real-world potential for 2 and 7 at detecting Fe^3+^ in two types of samples: tap water and mineral water. Both 2 and 7 demonstrated measurable reductions in *λ*_em_ at 420 nm in the presence of 50 μM and 100 μM Fe^3+^ (Fig. 13 for 7 and ESI S6† for 2).

**Fig. 14:**
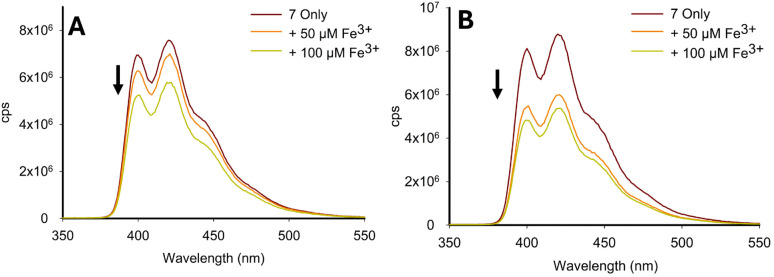
Fe^3+^-triggered “turn-off” response for 7 in pond water (Panel A). Fe^3+^-triggered “turn-off” response for 7 in river water (Panel B), *λ*_ex_ 250 nm. Solvent composition was 7 : 3 MeCN : H_2_O; cps is counts per second.

This study indicated that despite the reduction in the “turn-off” response observed in the competition assays (Fig. 12), the “turn-off” response was detectable in real world samples. Limit of detection (LoD) studies were performed in a 7 : 3 MeCN : H_2_O solution, and confirmed that 2 and 7 had LoD values of 2.12 μM and 3.41 μM, respectively (see ESI S8†). This confirmed that both pyrazoline 2 and pyrazole 7 can detect Fe^3+^ below the iron drinking water limit of the EPA and EU, and validates their application in real-world monitoring. These LoD are comparable with other Fe^3+^-specific fluorescence sensors. For example, Chattopadhyay *et al.* reported on a Fe^3+^-specific “turn-off” sensor with a LoD value of 3.5 μM,^33^ Goswami *et al.* reported on a Fe^3+^ “turn-on” sensor with a LoD of 2.9 μM,^34^ and Wang *et al.* reported on a Fe^3+^-specific colorimetric sensor with a LoD value of 1.0 μM^35^ (see ESI S8† for further Fe^3+^ LoD examples). An additional study was conducted with 7 using pond (Fig. 14A) and river (Fig. 14B) water samples with a measurable “turn-off” response with 50 μM and 100 μM Fe^3+^, respectively (see ESI S7† for similar study with 2). This confirmed that both 2 and 7 can operate in external water sources containing a range of interferences beyond the cations screened in the competition studies (Fig. 12), such as sediment and bacteria present in natural water sources.

**Fig. 15:**
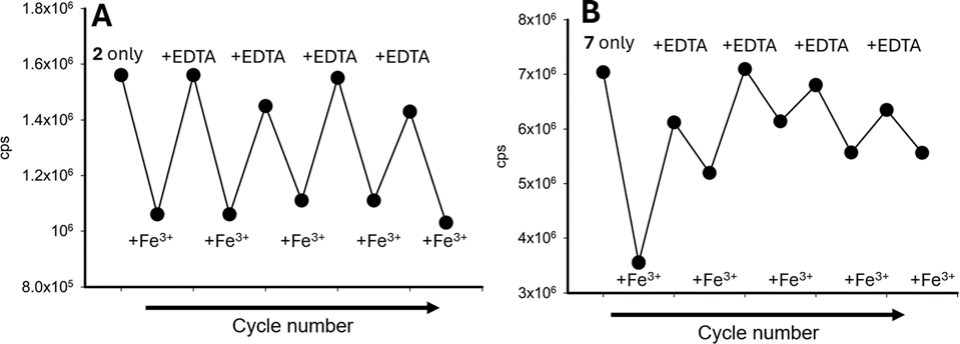
Repeatability study of the addition of 5.0 equivalents of Fe^3+^, followed by 5.0 equivalents of EDTA, repeated for 5 cycles in a 7 : 3 MeCN : H_2_O solution. (Panel A) is 2*λ*_ex_ 280 nm and (Panel B) is 7*λ*_ex_ 250 nm; cps is counts per second.

A reversibility study was conducted with 2 and 7 in the presence of several cycles of Fe^3+^, followed by EDTA, confirming that both sensors can be used multiple times for Fe^3+^ detection (Fig. 15A for 2 and Fig. 15B for 7).

**Fig. 16:**
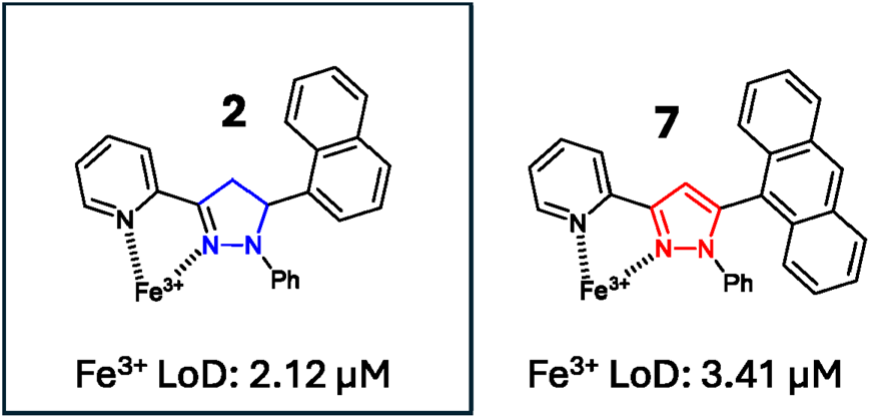
Proposed 1 : 1 binding mechanism of 2 and 7 with Fe^3+^ with the calculated limit of detection for Fe^3+^ in 7 : 3 MeCN : H_2_O solution. Pyrazoline 2 was selected as the lead compound from this study.

A proposed 1 : 1 binding mechanism of Fe^3+^ with sensors 2 and 7 is shown in Fig. 16, and agrees with the Job plot for 7 with Fe^3+^ (see ESI S11†) and the previously reported X-ray crystal structure complex of the pyrazole A analogue with Zn^2+^.^15^

## Conclusions

Seven novel polycyclic pyrazoline and pyrazoles were synthesised to determine how phenyl, naphthalene and anthracene units influence photophysical properties. Small modifications (for example, substitution of a methyl in 1 for a phenyl in 2) reversed the photophysical properties from “turn-on” to “turn-off”. 2 retained a good “turn-off” response in 7 : 3 MeCN : H_2_O solutions, and was selected for further analysis. The addition of a third aromatic ring to 2, resulting in 3, significantly disrupted *λ*_em_ and was detrimental to sensing. Pyrazoline 4 displayed minimum *λ*_em_ and was unsuitable for sensing applications. For the pyrazole series, 5 demonstrated a “turn-on” response at *λ*_em_ 310 nm to a variety of metals, whereas 6 displayed a “turn-off” response at *λ*_em_ 420 nm to a range of metals. Neither showed selectively to Fe^3+^, and were not suitable for sensing. 7 was the most useful pyrazole with complete quenching of *λ*_em_ at 420 nm on addition of 4.0 equivalents of Fe^3+^ in organic solutions. Lead sensors 2 and 7 were confirmed to display a Fe^3+^-triggered “turn-off” response in aqueous samples with the Fe^3+^ limit of detection values of 2.12 μM and 3.41 μM, respectively. Real-world analysis confirmed that 2 and 7 could detect Fe^3+^ in both tap water and mineral waters, and be useful for the industrial scale monitoring of Fe^3+^ in drinking water. 2 and 7 were also confirmed to display a “turn-off” response in pond and river waters, suggesting these sensors could be used for environmental monitoring of Fe^3+^ in external water sources. Further work is required to mitigate competing cations from influencing the fluorescence response. The introduction of additional chelation groups around the pyridine is a promising solution, as it has been shown to be highly beneficial for C and D.^17^ These results set a firm foundation for the development of future generations of improved phenyl substituted pyrazoline and pyrazole Fe^3+^-specific “turn-off” sensors that are purposely designed to operate in aqueous environments.

## Data availability

The data supporting this article have been included as part of the ESI.†

## Author contributions

Alexander Ciupa designed, synthesized, characterised, performed all spectroscopy studies, and authored the manuscript.

## Conflicts of interest

There are no conflicts to declare.

## Supplementary Material

RA-014-D4RA06457G-s001
