# Supplementary material for: Novel polycyclic “turn-on” and “turn-off” pyrazoline and pyrazole fluorescent sensors for selective real-world monitoring of Fe3+/Fe2+ in aqueous environments
Source: RSC Adv. 2024 Oct 31;14(47):34918–24. doi: 10.1039/d4ra06457g (PMC11526818; doi:10.1039/d4ra06457g)
Supplement: RA-014-D4RA06457G-s001 [file RA-014-D4RA06457G-s001.pdf]

Supplementary Information (SI) for RSC Advances.
This journal is © The Royal Society of Chemistry 2024

Electronic Supplementary Information (ESI)

Novel polycyclic “turn on” and “turn off” pyrazoline and pyrazole fluorescent sensors selective for Fe^{3+}/Fe^{2+} in aqueous environments for real-world monitoring

Alexander Ciupa*

General Experimental

Chemicals, solvents and reagents were purchased from commercial sources and used without further purification. PE refers to petroleum ether, bp 40-60 °C. Spectroscopy was performed with CHROMASOLV® gradient grade acetonitrile for HPLC, ≥99.9%, from Sigma-Aldrich.

The metal complexes used in this study were: LiCl, NaCl, KCl, $CaCl_2$, $MgCl_2$, $CuCl_2$, $CuSO_4$, $Cu(OAC)_2$, $NiCl_2$, $ZnCl_2$, $CdCl_2$, $RuCl_3$, $CoCl_2$, $MnCl_2$, $PbCl_2$, $ZnCl_2$, $FeSO_4$ and $FeCl_3$.

TLCs were carried out on Merck Aluminium backed TLC plates Silica Gel 60 F254 and viewed using UV light of wavelength 254 nm. Merck Silica Gel (0.040-0.063 mm) was used for column chromatography. Compounds were loaded as an oil, CH_2Cl_2 solution or dry loaded by adsorption onto silica.

NMR spectra were obtained on a Bruker Avance III (400 MHz) spectrometer and processed via TopSpin® software. The chemical shifts are recorded in parts per million (ppm) with reference to tetramethylsilane. The coupling constants J are quoted to the nearest 0.5 Hz and are not corrected.

High resolution Mass spectroscopy was performed on Bruker Quadrupole Time-of-Flight (qToF) mass spectrometer.

UV/Vis spectroscopy was performed on an Agilent Cary5000 in quartz cuvettes with a 1 cm pathlength using HPLC grade MeCN, 250-500 nm range with 0.2 sec dwell time. Detector switchover occurred at 350 nm.

FTIR spectroscopy was performed on a Bruker VERTEX 70 spectrometer.

Fluorescence spectroscopy was performed on an Edinburgh Instruments FLS1000 with a xenon excitation source, 2 nm bandwidths for both excitation and emission monochromator, scan speed of 1 nm and dwell time of 0.2 sec. Fluorescence quartz cuvettes with a 1 cm pathlength were used throughout with HPLC grade MeCN. Quantum yields were determined using the absolute method and use of a Edinburgh instruments integrating sphere, https://www.edinst.com/wp-content/uploads/2016/02/FLS980-Series-Reference-Guide-Integrating-Sphere.pdf

A 100 Watt 365 nm Analytikjena High intensity UV lamp was used to image the sensors in cuvettes with 5.0 equivalent indicated metal, sensor concentration was 20 μM, solvent was MeCN.

All figures were plotted using SigmaPlot® 14.5 software.

General Synthesis (S1)

Synthesis of chalcones **C1**-**C2**

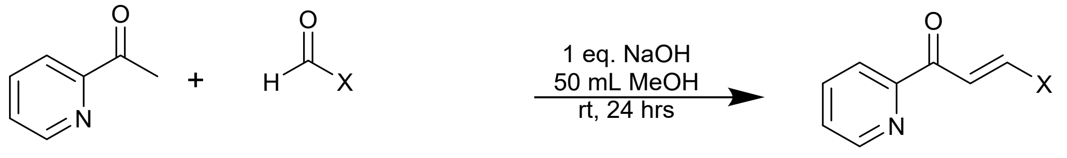

Using a method adapted from a previous synthesis (*RSC Adv.*, 2017, **7**, 44272), 5.0 mmol 2-acetylpyridine was added to a stirred solution of 5.0 mmol aldehyde (1-naphthaldehyde or 9-anthraldehyde) in MeOH followed by the addition of 5.0 mmol NaOH and stirring continued. After 24 hours the solvent was removed under reduced pressure and the residue was filtered, washed with copious amounts of cold H_2O and collected and dried to afford the desired chalcone without further purification.

Synthesis of 1-4

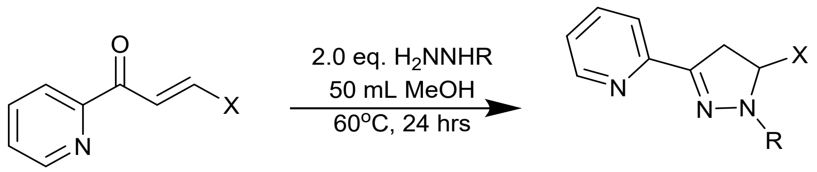

Using a method adapted from a previous synthesis (*RSC Adv.,* 2024, **14**, 3519), the required hydrazine (H_2NNHMe or H_2NNHPh) 2.0 mmol was added to a stirred solution of the required chalcone (**C1** or **C2**) 1.0 mmol in MeOH at 60°C. After 24 hours the solvent was removed under reduced pressure to afford an oil which was extracted into 100 mL ethyl acetate and washed with 3 x 50 mL water. The ethyl acetate was removed under reduced pressure to afford an oil which was further purified by column chromatography using an 8:2 PE: ethyl acetate mixture to give the desired pyrazoline.

Synthesis of 5-7

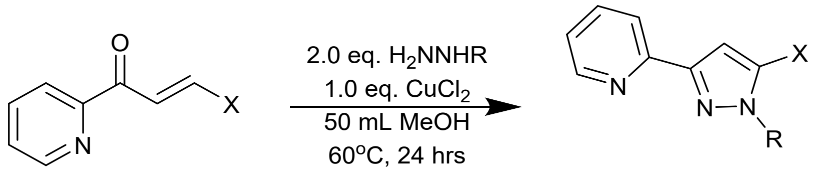

Using a method from the literature (*New J. Chem.,* 2024, **48**, 13900), the required hydrazine (H_2NNHMe or H_2NNHPh) 2.0 mmol was added to a stirred solution of required chalcone (**C1** or **C2**) 1.0 mmol in 50 mL MeOH at 60°C followed by the addition of $CuCl_2$ (1.0 mmol). After 24 hrs the solution was removed under reduced pressure, the residue resuspended in 100 mL of a saturated EDTA solution and extracted with ethyl acetate (3 x 50 mL). The ethyl acetate layers were combined, and solvent removed under reduced pressure. The residue was then purified by column chromatography using PE: ethyl acetate (80:20) to afford the required pyrazole.

Synthesis of (E)-3-(naphthalen-1-yl)-1-(pyridin-2-yl)prop-2-en-1-one (**C1**)

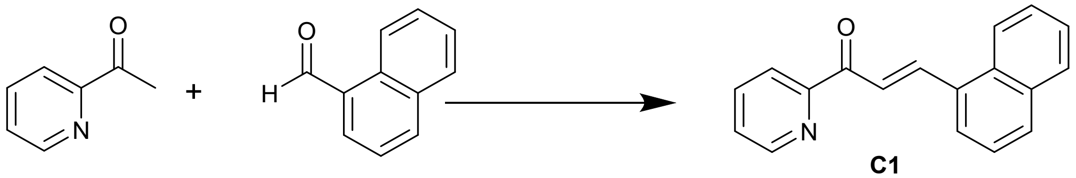

C1

Yield 1.21g (93%);

Vmax (Solid)/cm^{-1} 1854, 1533, 1427and 1106;

^{1}H NMR δ_H (400 MHz; $CDCl_3$) 7.52-7.64 (5 H, m, CH), 7.91-7.97 (3 H, m, CH), 8.09 (1 H, d, *J* = 7.2 Hz, CH), 8.27 (1 H, d, *J* = 8 Hz, CH), 8.37 (1 H, d, *J* = 8.4 Hz, CH), 8.43 (1 H, d, *J* = 16.0 Hz, CH), 8.79 (1 H, m, CH) and 8.82 (1 H, d, *J* = 16.0 Hz, CH);

^{13}C NMR δ_c (400 MHz; $CDCl_3$) 121.8, 123.0, 123.3, 123.5, 125.1, 125.5, 125.6, 126.2, 126.9, 131.0, 132.0, 132.4, 133.8, 137.1, 141.4, 148.0, 154.3 and 189.4;

HRMS m/z (qToF) Found 260.1104 (M+H$^+$). $C_{18}H_{14}NO$ requires 260.1075.

Synthesis of (E)-3-(anthracen-9-yl)-1-(pyridin-2-yl)prop-2-en-1-one (**C2**)

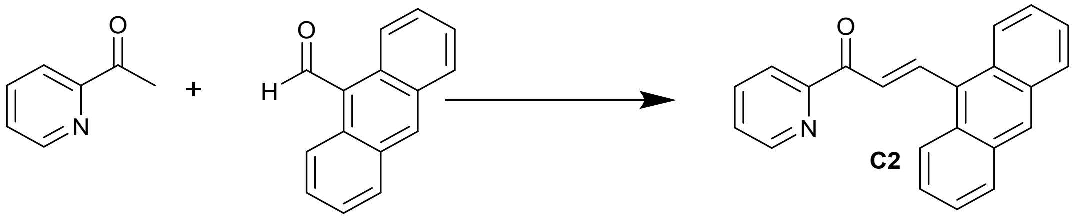

C2

Yield 1.15g (75%);

Vmax (Solid)/cm^{-1} 1717, 1531, 1426, 1381 and 1163;

^{1}H NMR δ_H (400 MHz; $CDCl_3$) 7.52-7.56 (5 H, m, CH), 7.94-7.95 (1 H, m, CH), 8.45-8.07 (2 H, m, CH), 8.29-8.33 (2 H, m, CH), 8.39-8.41 (2 H, m, CH), 8.50 (1 H, s, CH), 8.72-8.74 (1 H, m, CH) and 8.96 (1 H, d, *J* = 16.0 Hz, CH);

^{13}C NMR δ_c (400 MHz; $CDCl_3$) 122.6, 123.1, 123.6, 125.1, 125.4, 125.5, 125.6, 126.7, 127.0, 127.3, 128.4, 128.5, 128.8, 129.1, 129.3, 129.8, 130.2, 130.4, 134.1, 136.8, 154.2 and 189.4;

HRMS m/z (qToF) Found 310.1255 (M+H$^+$). $C_{22}H_{16}NO$ requires 310.1232.

Above data consistent with spectra from *RSC Adv.,* 2017, **7**, 44272.

Synthesis of 2-(1-methyl-5-(naphthalen-1-yl)-4,5-dihydro-1H-pyrazol-3-yl)pyridine (**1**)

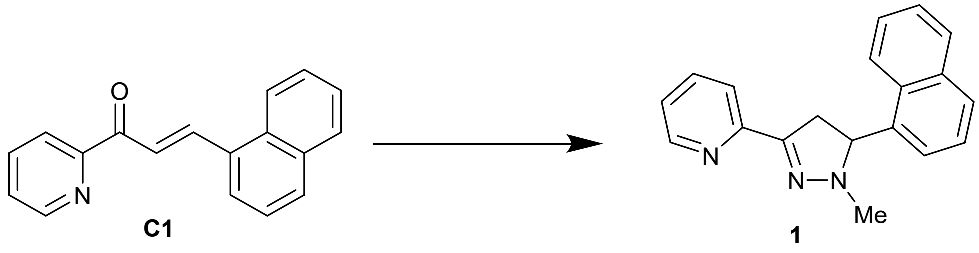

Yield 0.065g oil (23%);

Vmax (film)/cm^{-1} 1855, 1548, 1427, 1381 and 1166;

^{1}H NMR δ_H (400 MHz; CDCl$_3$) 3.05-3.13 (4 H, m, CH$_3$), 4.06 (1 H, m, CH), 4.95 (1 H, m, CH), 7.21 (1 H, m, CH), 7.53- 7.55 (3 H, m, CH), 7.68-7.07 (1 H, m, CH), 7.26-7.38 (2 H, m, CH), 7.85-7.93 (1 H, m, CH), 7.94-7.97 (1 H, m, CH), 8.07-8.09 (1 H, m, CH) and 8.57-8.59 (1 H, m, CH);

^{13}C NMR δ_c (400 MHz; CDCl$_3$) 41.8, 120.5, 122.7, 123.4, 124.0, 125.4, 125.7, 126.1, 126.4, 128.1, 128.6, 128.9, 131.4, 134.0, 136.1, 136.2, 149.2, 150.2 and 152.1;

HRMS m/z (qToF) Found 288.1476 (M+H$^+$). $C_{19}H_{18}$N3 requires 288.1501.

Synthesis of 2-(5-(naphthalen-1-yl)-1-phenyl-4,5-dihydro-1H-pyrazol-3-yl)pyridine (**2**)

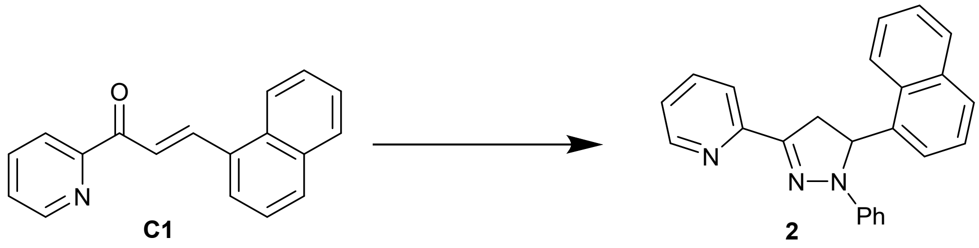

Yield 0.093g oil (26%);

Vmax (film)/cm^{-1} 1704, 1565, 1443, 1237 and 1198;

^{1}H NMR δ_H (400 MHz; CDCl$_3$) 3.34-3.40 (1 H, m, CH), 4.21-4.26 (1 H, m, CH), 6.05-6.84 (1 H, m, CH), 6.82-6.85 (1 H, m, CH), 7.10-7.18 (2 H, m, CH), 7.18-7.23 (3 H, m, CH), 7.35-7.38 (2 H, m, CH), 7.58-7.60 (3 H, m, CH), 7.07-7.74 (1 H, m, CH), 7.78-7.81 (1 H, m, CH), 8.12-8.14 (1 H, m, CH), 8.19-8.21 (1 H, m, CH) and 8.51-8.52 (1 H, m, CH);

^{13}C NMR δ_c (400 MHz; CDCl$_3$) 42.2, 113.5, 119.6, 120.6, 122.7, 123.1, 125.8, 126.4, 128.0, 128.9, 129.0, 129.2, 129.9, 134.4, 136.0, 136.7, 144.3, 147.1, 148.6, 149.1, 152.1 and 152.1;

HRMS m/z (qToF) Found 338.1620 (M+H$^+$). $C_{23}H_{20}N_3$ requires 338.1657.

Synthesis of 2-(5-(anthracen-9-yl)-1-methyl-4,5-dihydro-1H-pyrazol-3-yl)pyridine (**3**)

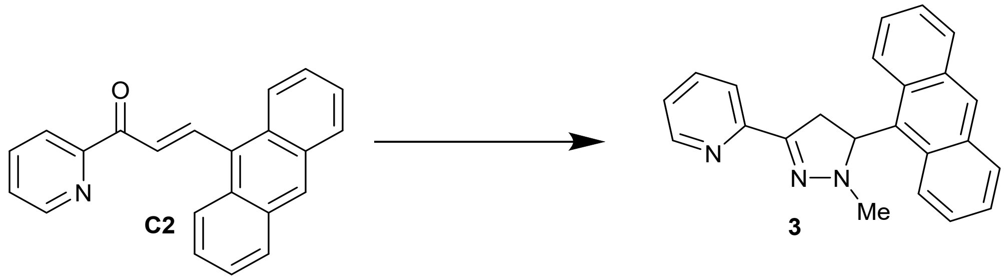

Yield 0.024g oil (7%);

Vmax (film)/cm^{-1} 1856, 1550, 1444 and 1192;

^{1}H NMR δ_H (400 MHz; $CDCl_3$) 2.82 (3 H, s, CH_3), 3.57- 3.65 (1 H, m, CH), 3.78-3.81 (1 H, m, CH), 5.66-5.72 (1 H, m, CH), 7.15-7.16 (1 H, m, CH), 7.33-7.46 (5 H, m, CH), 7.63-7.66 (1 H, m, CH), 7.93-7.99 (2 H, m, CH), 8.27-8.29 (1 H, m, CH), 8.40 (1 H, s, CH), 8.51- 8.52 (1 H, m, CH) and 8.73-8.74 (1 H, m, CH);

^{13}C NMR δ_c (400 MHz; $CDCl_3$) 41.5, 68.0, 120.6, 122.5, 122.7, 124.8, 125.0, 125.1, 125.6, 126.1, 126.4, 126.7, 127.3, 128.5, 129.4, 129.5, 129.6, 129.8, 131.0, 131.2, 134.1, 136.1, 149.3, 149.6 and 152.3;

HRMS m/z (qToF) Found 350.1627 (M+H$^+$). $C_{24}H_2N_3$ requires 350.1657.

Synthesis of 2-(5-(anthracen-9-yl)-1-phenyl-4,5-dihydro-1H-pyrazol-3-yl)pyridine (**4**)

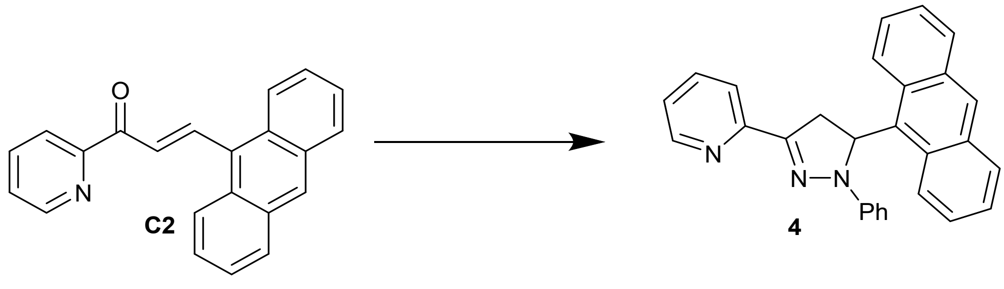

Yield 0.061g oil (15%);

Vmax (film)/cm^{-1} 1854, 1546, 1427 and 1237;

^{1}H NMR δ_H (400 MHz; $CDCl_3$) 3.66-3.74 (1 H, m, CH), 4.22-4.30 (1 H, m, CH), 6.70 (1 H, m, CH), 7.00 (3 H, m, CH), 7.36-7.39 (1 H, m, CH), 7.41-7.57 (2 H, m, CH), 7.60-7.66 (7 H, m, CH), 7.80 (1 H, m, CH), 8.01 (1 H, m, CH), 8.13 (2 H, m, CH), 8.31 (1 H, m, CH), 8.53 (2 H, m, CH) and 8.58 (1 H, m, CH);

^{13}C NMR δ_c (400 MHz; $CDCl_3$) 42.1, 61.3, 113.8, 119.9, 120.9, 122.3, 122.8, 124.2, 124.9, 125.1, 126.3, 127.0, 128.5, 128.8, 129.2, 129.4, 129.5, 129.0, 131.7, 132.2, 136.0, 145.3, 149.3 and 152.2;

HRMS m/z (qToF) Found 400.1774 (M+H$^+$). $C_{28}H_{22}N_3$ requires 400.1814.

Synthesis of 2-(1-methyl-5-(naphthalen-1-yl)-1H-pyrazol-3-yl)pyridine (**5**)

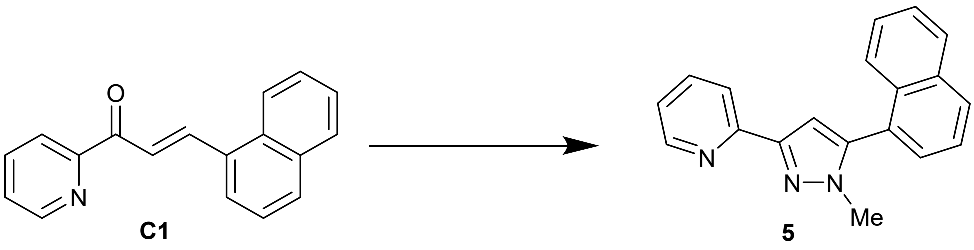

Yield 0.014g oil (5%);

Vmax (film)/cm^{-1} 1711, 1547 and 1190;

^{1}H NMR δ_H (400 MHz; $CDCl_3$) 4.03 (3 H, s, CH_3), 6.28-6.30 (1 H, m, CH), 6.49-6.53 (1 H, m, CH), 6.70 (1 H, s, CH), 6.26-7.63 (6 H, m, CH), 7.92-7.95 (3 H, m, CH), 8.05-8.06 (1 H, m, CH), 8.21- 8.22 (1 H, m, CH) and 8.92-8.94 (1 H, m, CH);

^{13}C NMR δ_c (400 MHz; $CDCl_3$) 39.5, 63.7, 107.4, 122.5, 122.9, 123.7, 125.4, 125.8, 126.2, 126.3, 127.0, 128.3, 131.4, 134.0, 136.8, 142.1, 149.3, 149.8 and 150.0;

HRMS m/z (qToF) Found 286.1301 (M+H$^+$). $C_{19}H_{16}N_3$ requires 286.1344.

Synthesis of 2-(5-(anthracen-9-yl)-1-methyl-1H-pyrazol-3-yl)pyridine (**6**)

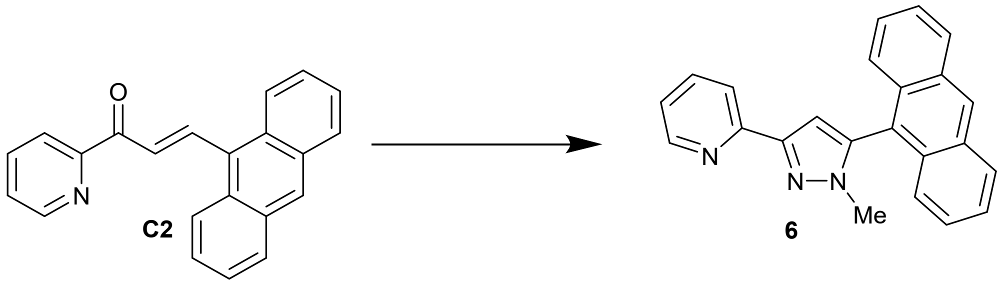

Yield 0.024g oil (7%);

Vmax (film)/cm^{-1} 1714, 1559, 1443 and 1243;

^{1}H NMR δ_H (400 MHz; $CDCl_3$) 3.95 (3 H, s, CH_3), 6.09-6.11 (1 H, m, CH), 6.41-6.44 (1 H, m, CH), 6.69 (1 H, s, CH), 7.29-7.31 (2 H, m, CH), 7.38-7.49 (6 H, m, CH), 7.98-8.00 (2 H, m, CH) and 8.50 (1H, s, CH);

^{13}C NMR δ_c (400 MHz; $CDCl_3$); 59.4, 103.6, 110.5, 113.6, 115.7, 116.8, 119.9, 121.8, 125.4, 125.9, 126.3, 128.2, 128.7, 131.6, 131.9, 134.2 and 138.6;

HRMS m/z (qToF) Found 336.1490 (M+H$^+$). $C_{23}H_{18}N_3$ requires 336.1501.

Synthesis of 2-(5-(anthracen-9-yl)-1-phenyl-1H-pyrazol-3-yl)pyridine (**7**)

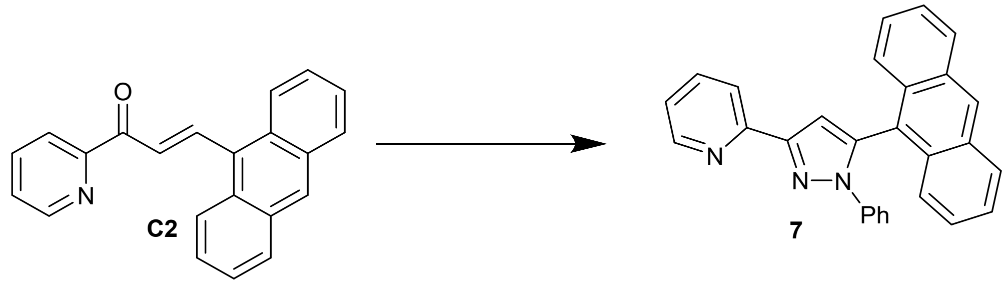

Yield 0.094g oil (24%);

Vmax (film)/cm^{-1} 1702. 1542, 1445 and 1167;

^{1}H NMR δ_H (400 MHz; $CDCl_3$) 6.99-7.01 (3 H, m, CH), 7.19-7.21 (2 H, m, CH), 7.21-7.29 (1 H, m, CH), 7.31-7.47 (5 H, m, CH), 7.81-7.85 (3 H, m, CH), 8.02-8.04 (2 H, m, CH), 8.26-8.29 (1 H, m, CH), 8.56 (1 H, s, CH) and 8.73-8.74 (1 H, m, CH);

^{13}C NMR δ_c (400 MHz; $CDCl_3$) 110.0, 120.5, 122.8, 123.4, 124.6, 125.4, 125.8, 126.6, 127.1, 128.5, 128.6, 128.9, 131.1, 131.2, 136.7, 140.0, 140.9, 149.6, 152.2 and 152.4;

HRMS m/z (qToF) Found 398.1610 (M+H$^+$). $C_{28}H_{20}N_3$ requires 398.1657.

NMR Spectra (S2)

E)-3-(naphthalen-1-yl)-1-(pyridin-2-yl)prop-2-en-1-one (**C1**)

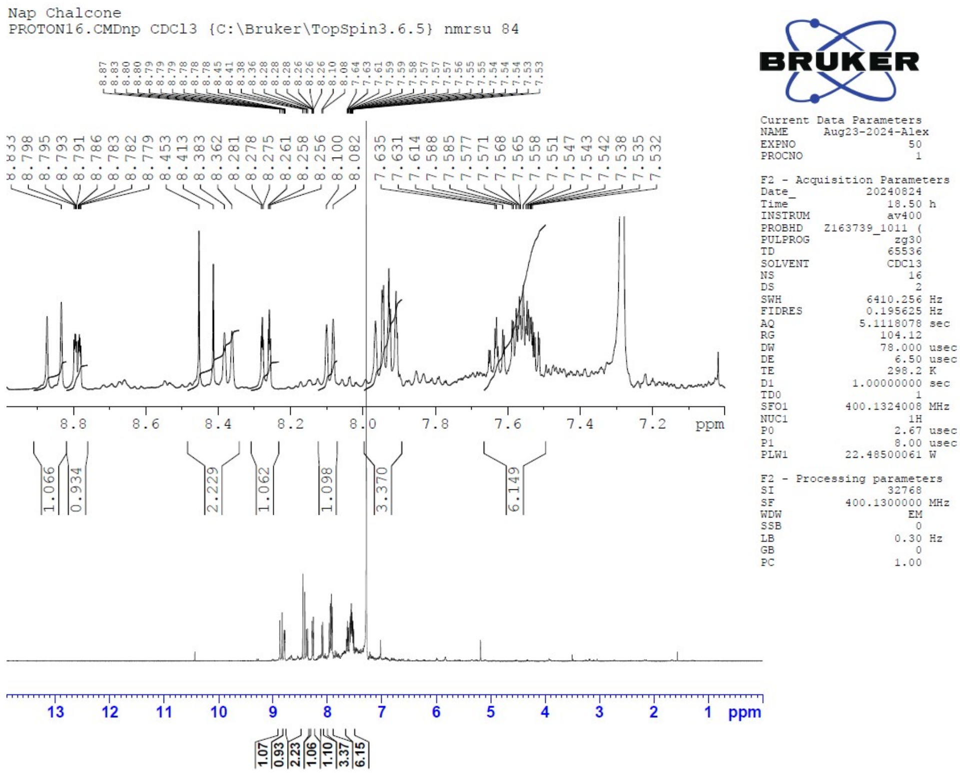

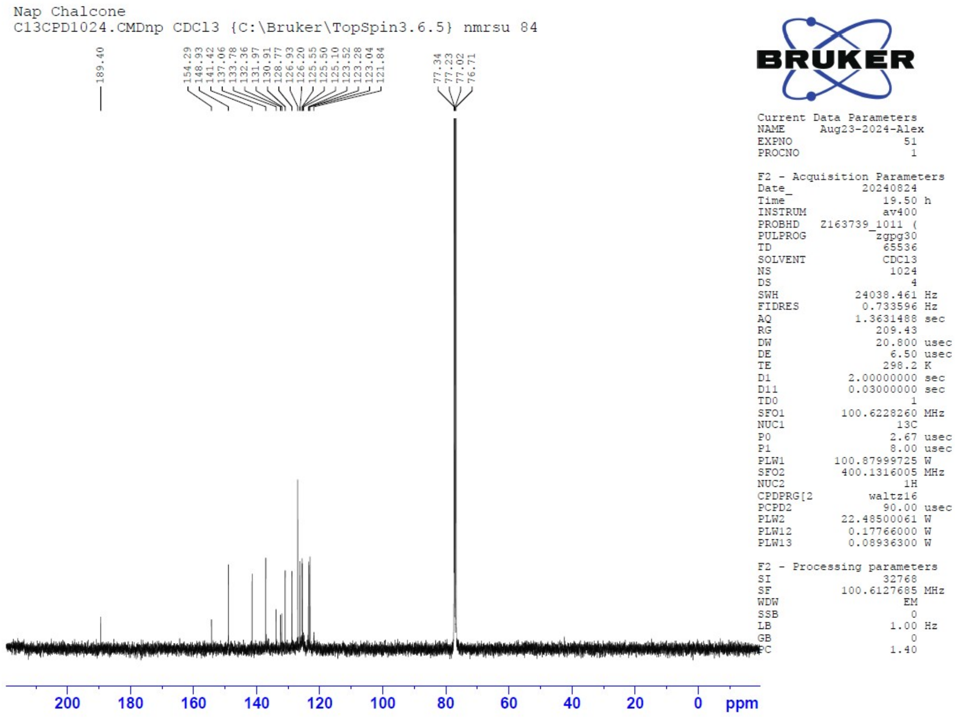

(E)-3-(anthracen-9-yl)-1-(pyridin-2-yl)prop-2-en-1-one (**C2**)

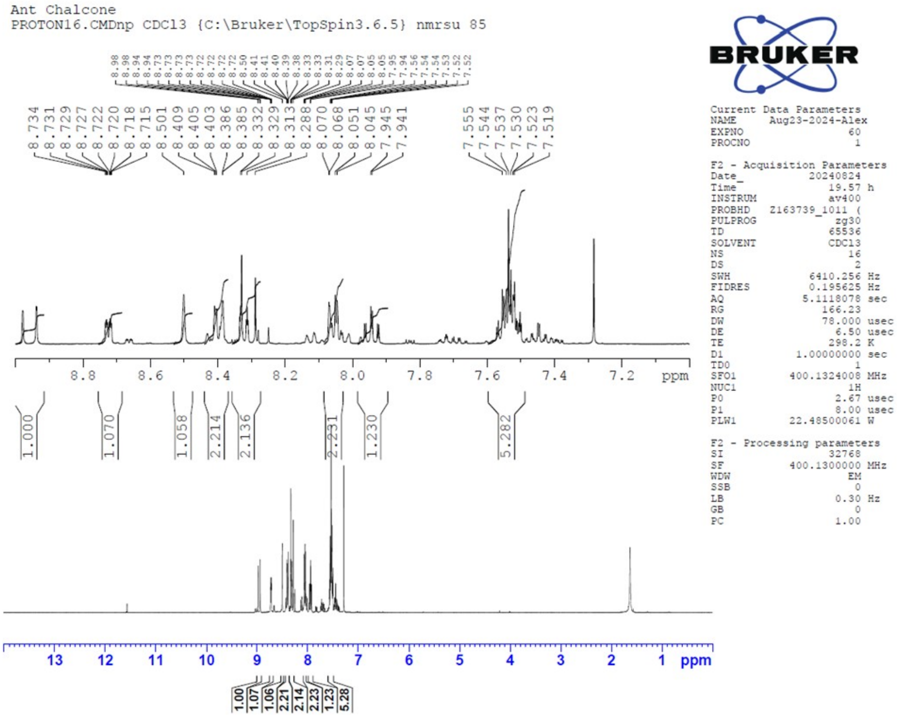

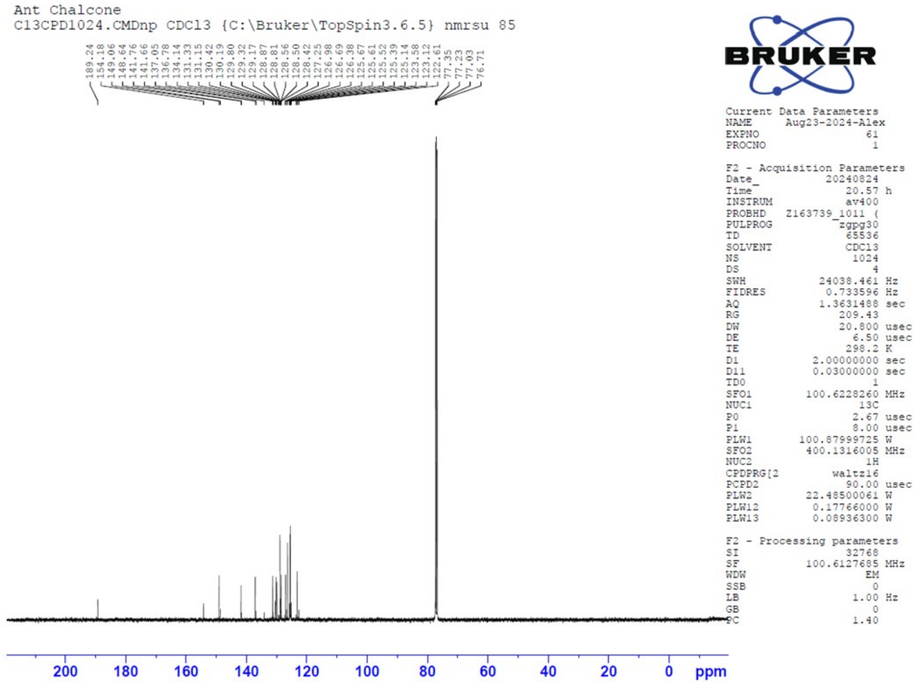

2-(1-methyl-5-(naphthalen-1-yl)-4,5-dihydro-1H-pyrazol-3-yl)pyridine (**1**)

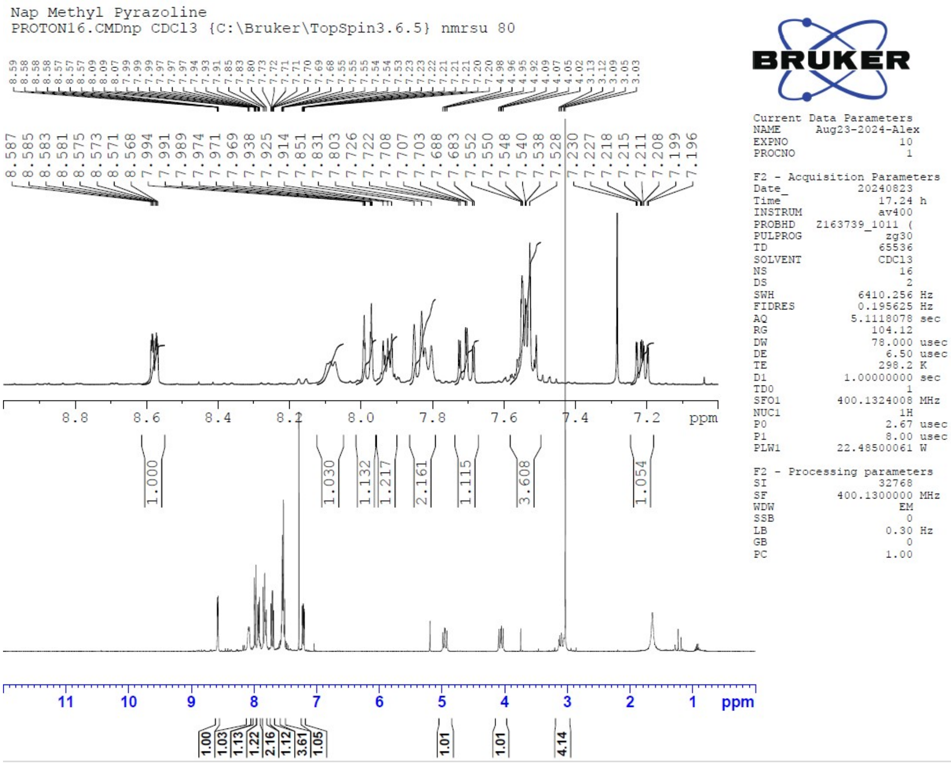

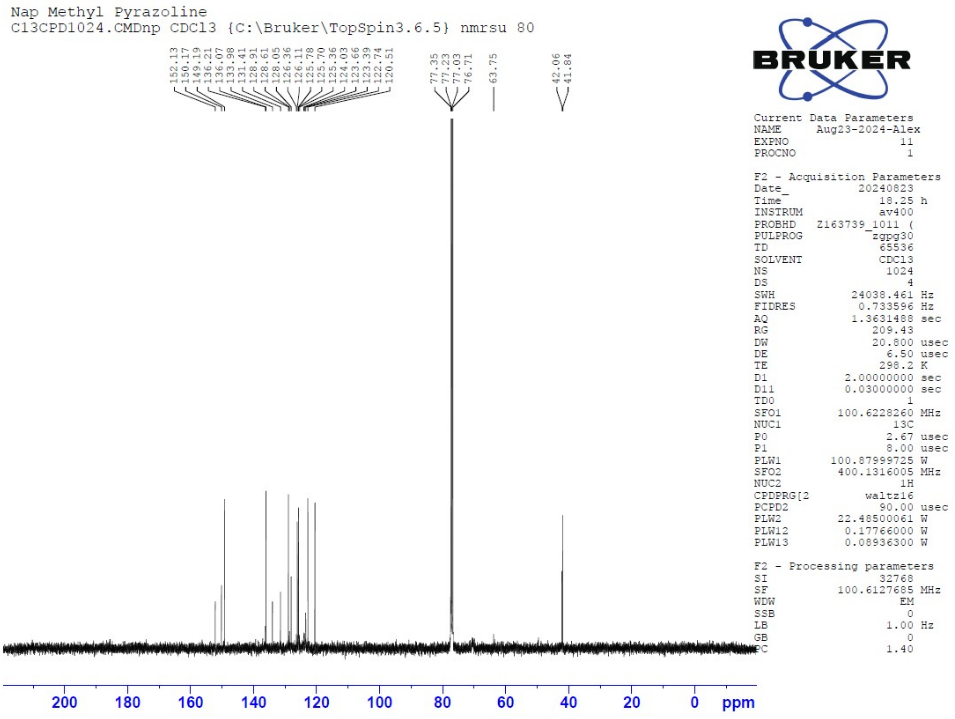

2-(5-(naphthalen-1-yl)-1-phenyl-4,5-dihydro-1H-pyrazol-3-yl)pyridine (**2**)

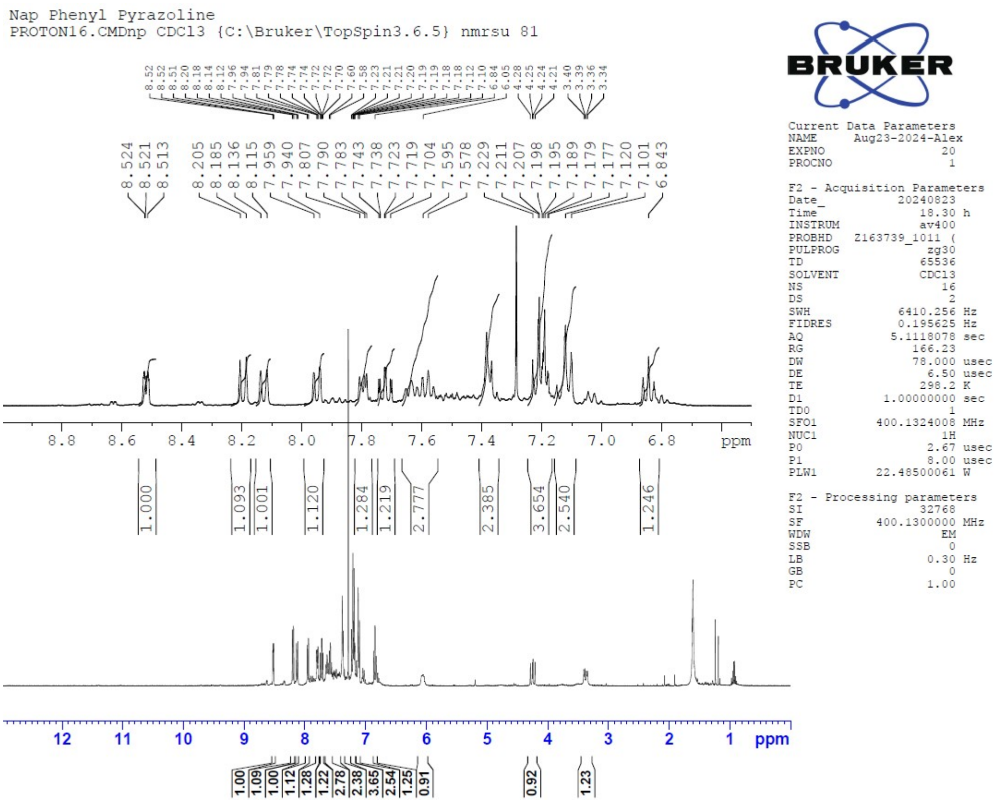

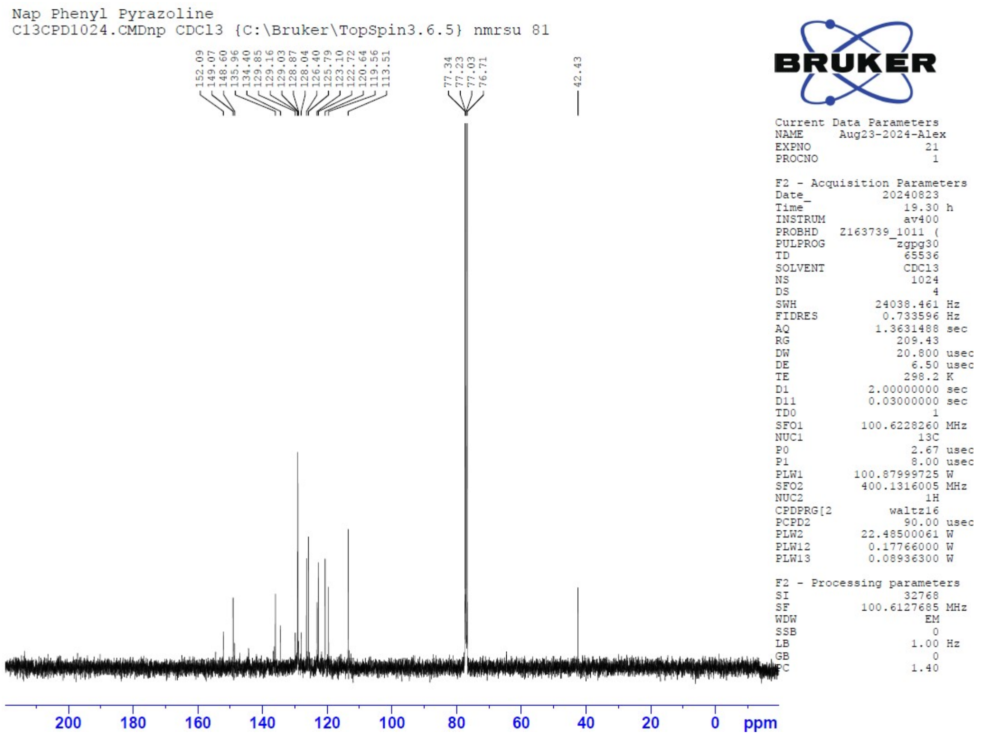

2-(5-(anthracen-9-yl)-1-methyl-4,5-dihydro-1H-pyrazol-3-yl)pyridine (**3**)

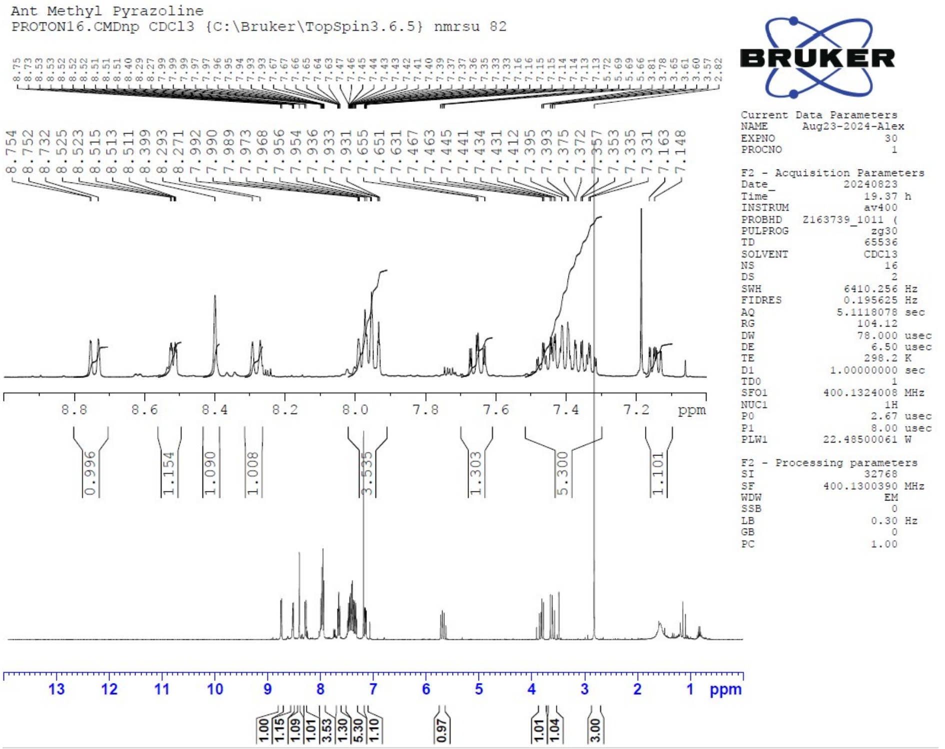

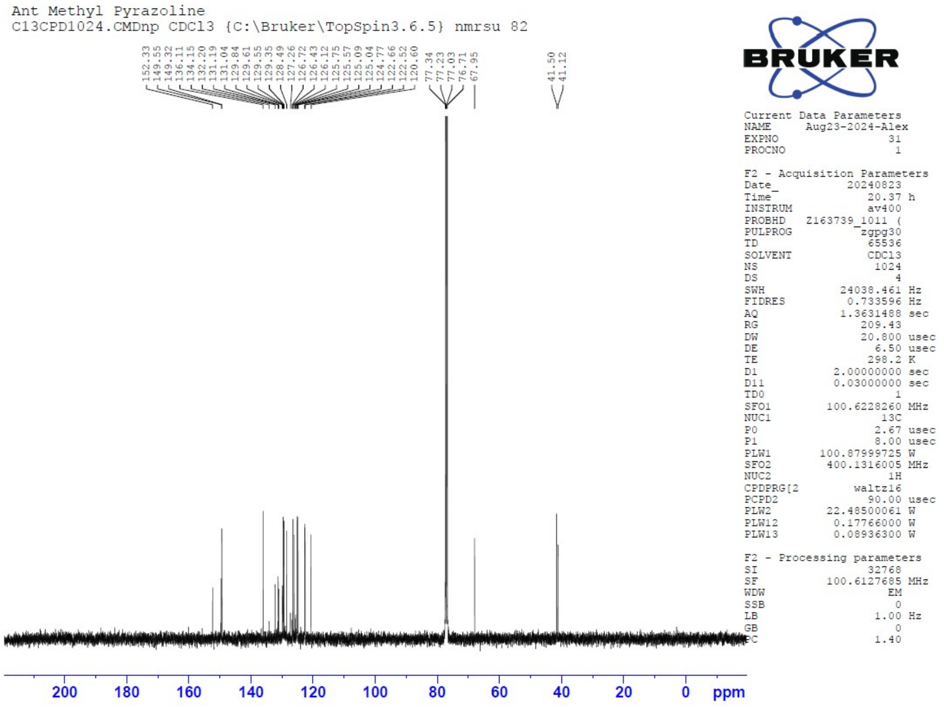

2-(5-(anthracen-9-yl)-1-phenyl-4,5-dihydro-1H-pyrazol-3-yl)pyridine (**4**)

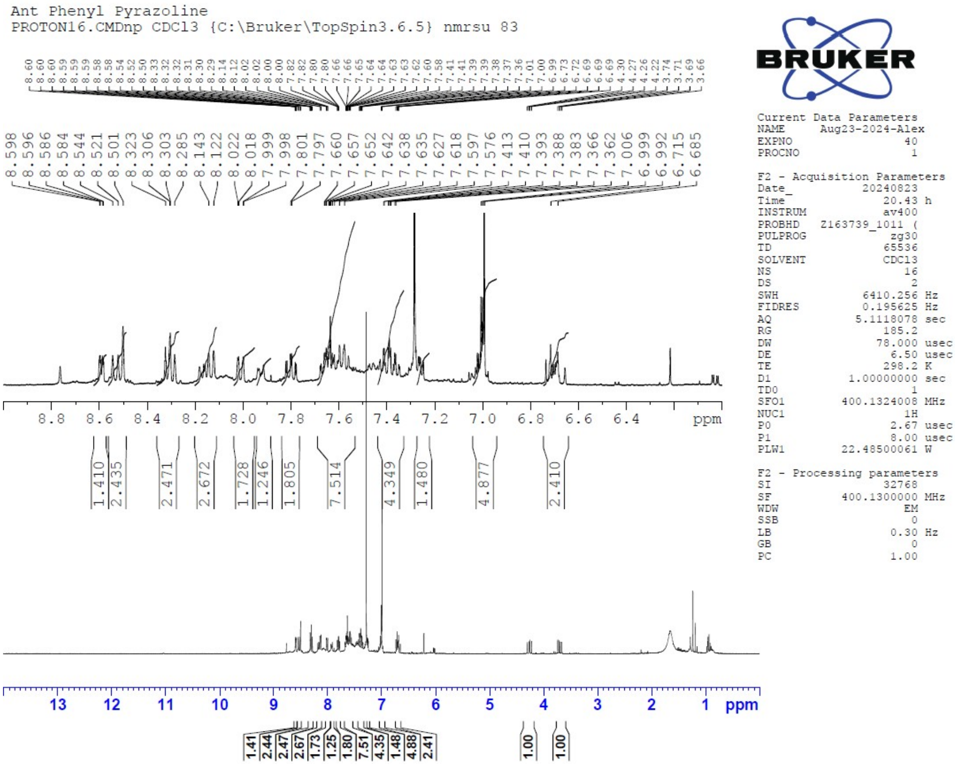

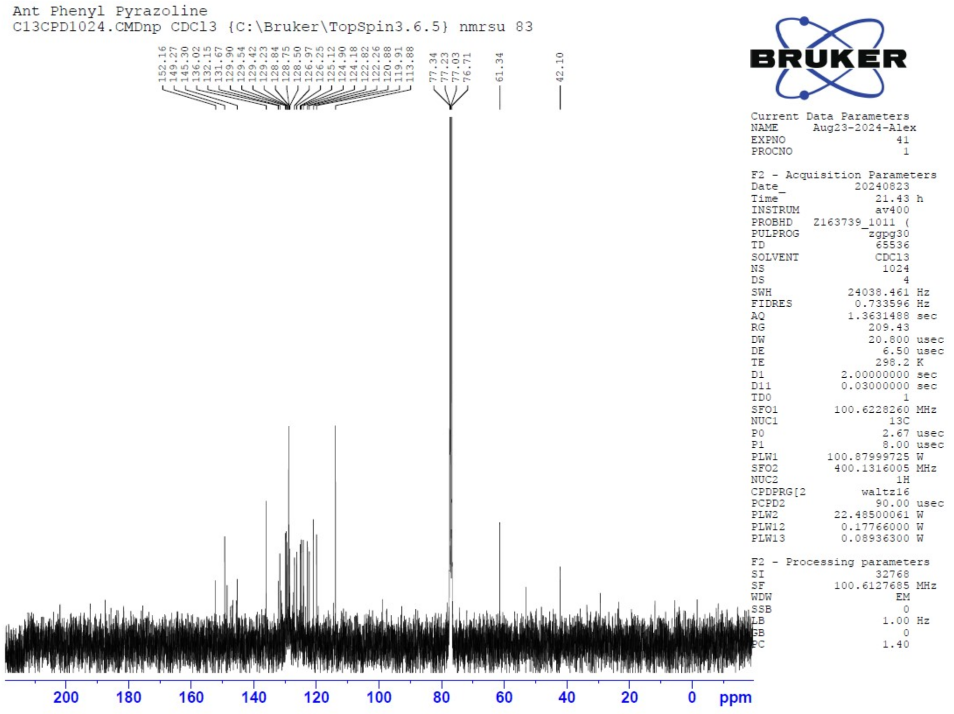

Synthesis of 2-(1-methyl-5-(naphthalen-1-yl)-1H-pyrazol-3-yl)pyridine (**5**)

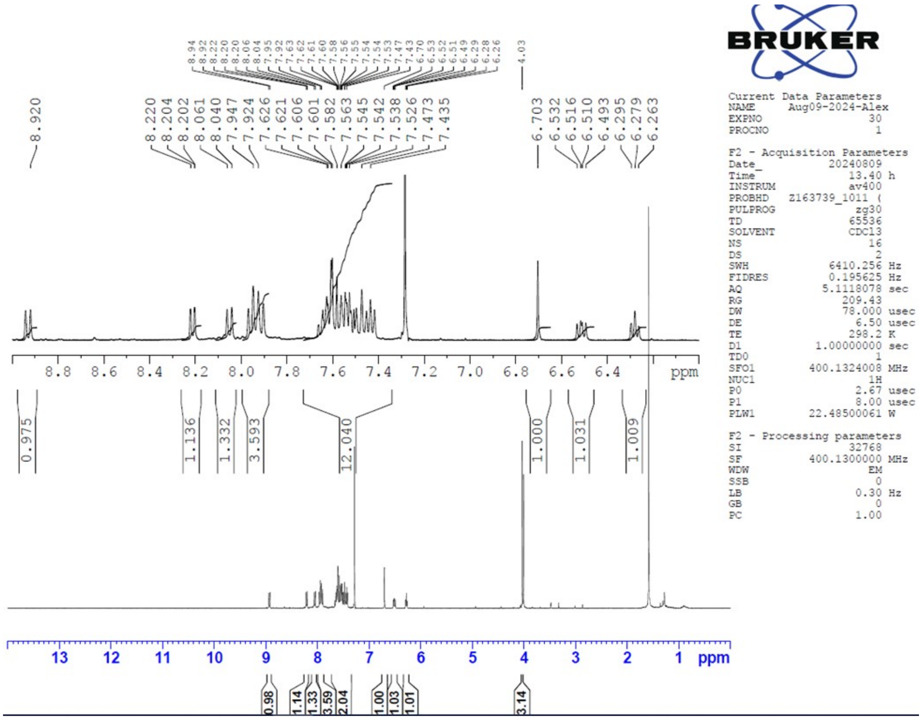

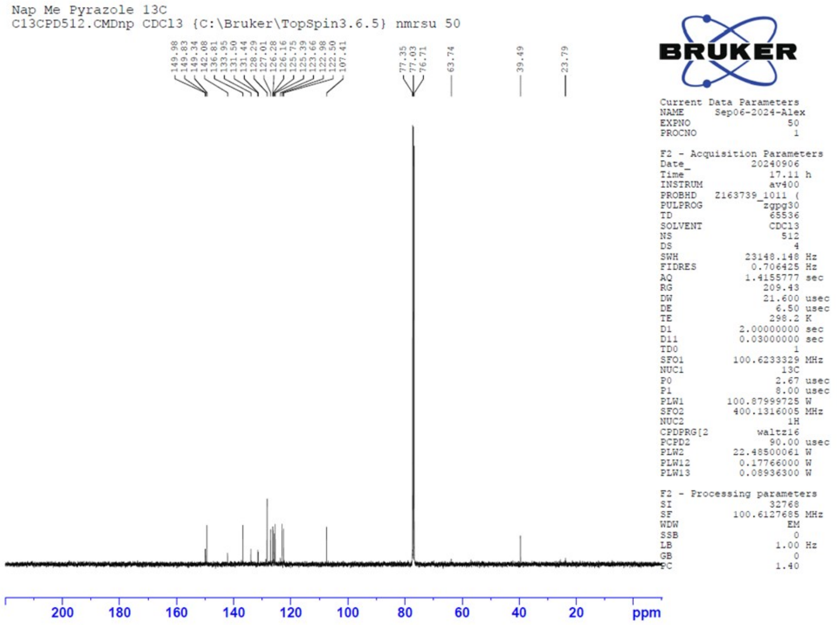

Synthesis of 2-(5-(anthracen-9-yl)-1-methyl-1H-pyrazol-3-yl)pyridine (**6**)

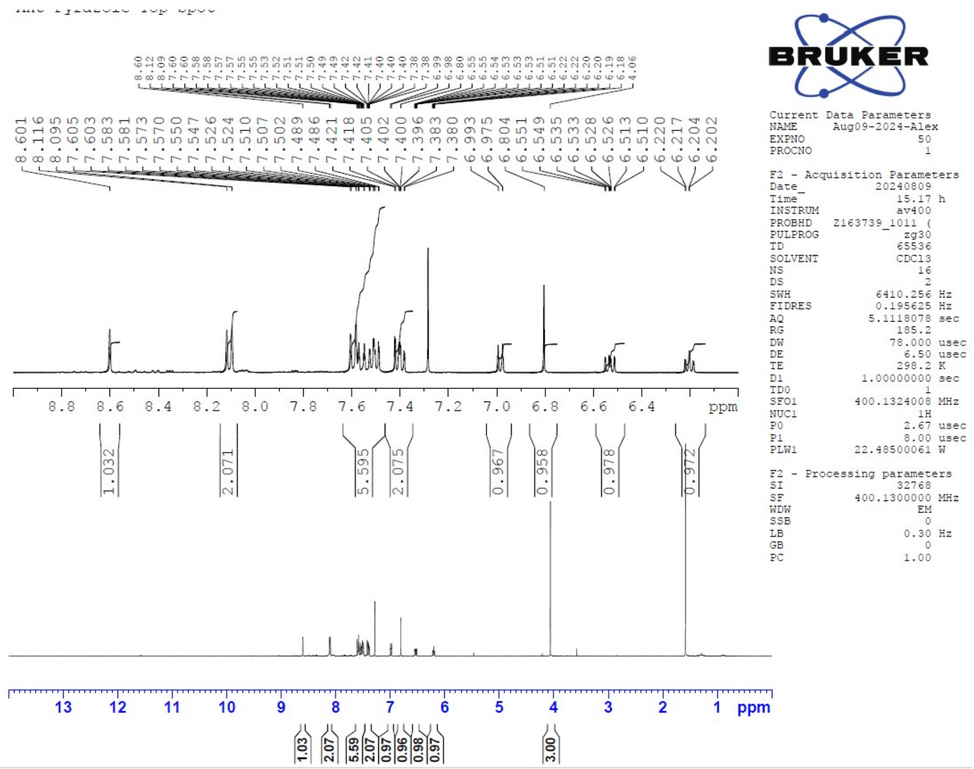

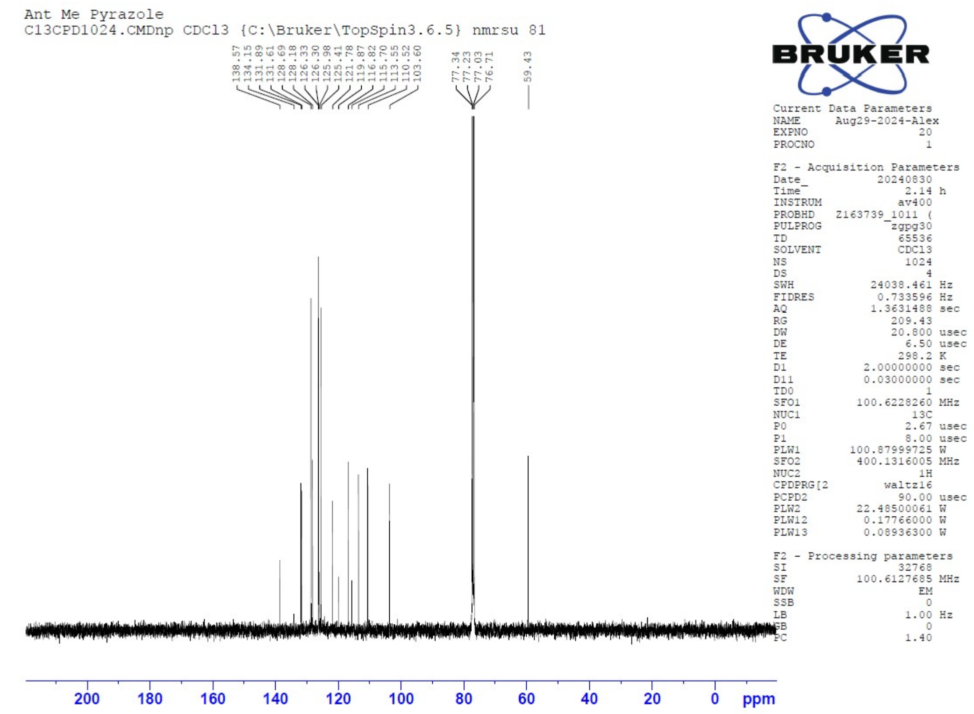

Synthesis of 2-(5-(anthracen-9-yl)-1-phenyl-1H-pyrazol-3-yl)pyridine (**7**)

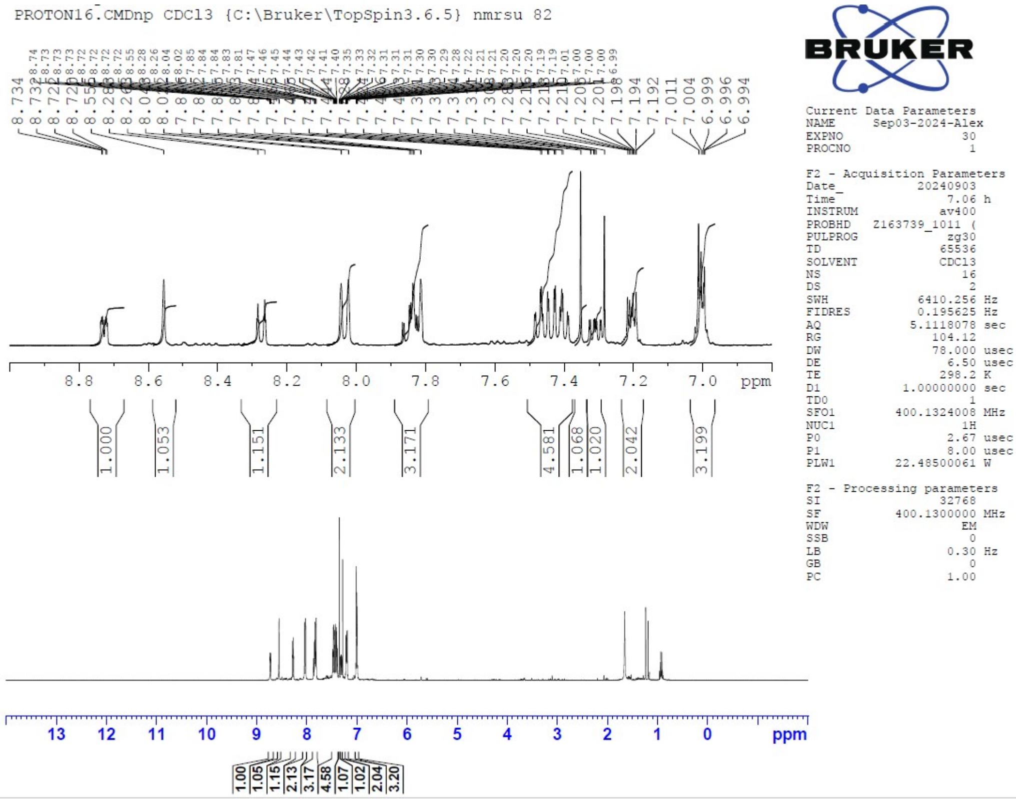

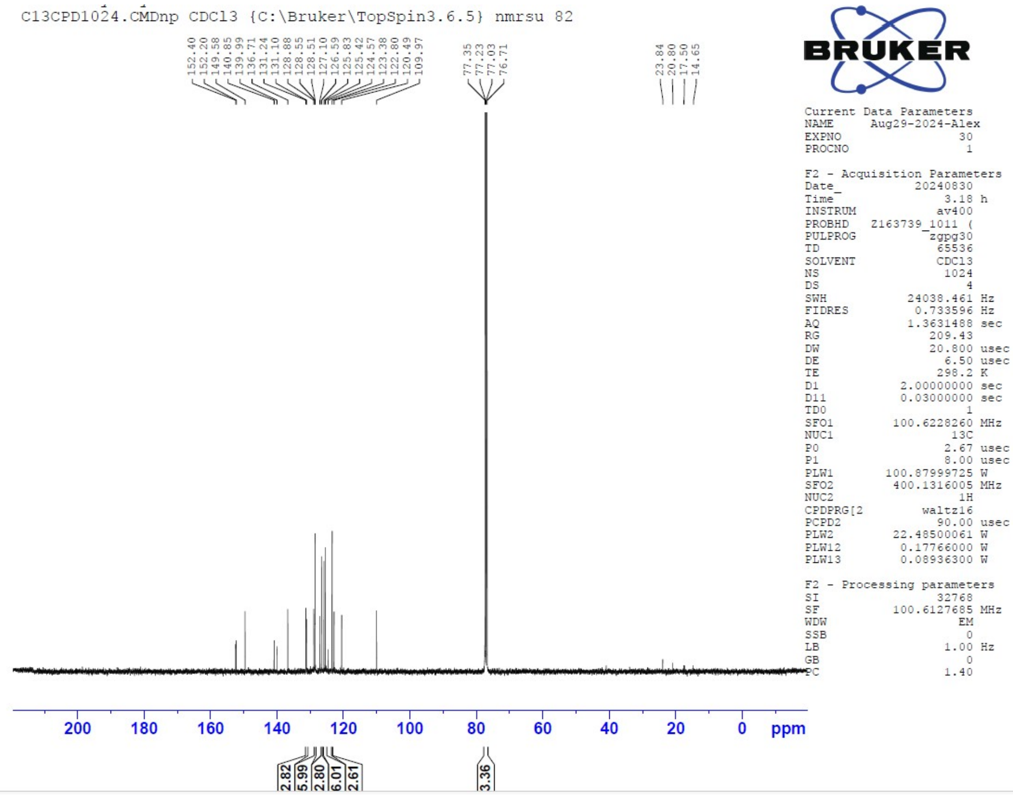

^{1}H NMR Experiments (S3)

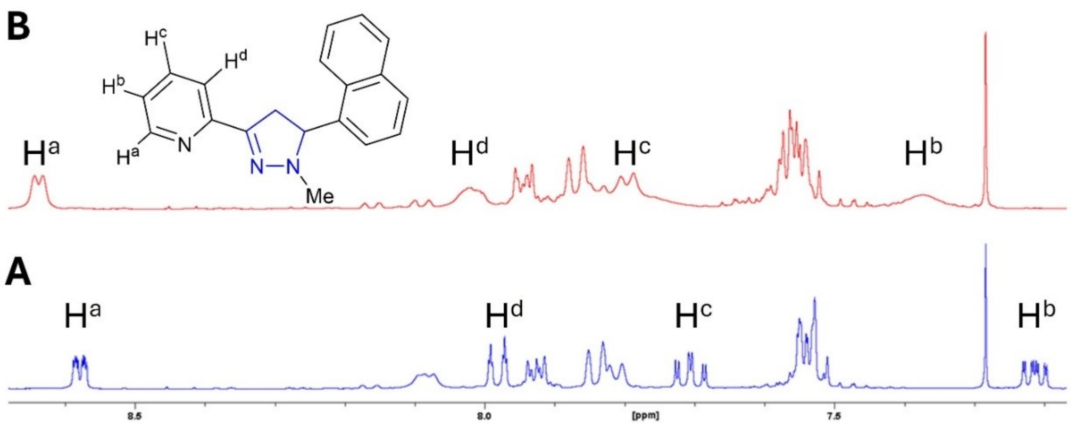

Partial ^{1}H NMR spectra for **1** only A (20 µM, $CDCl_3$) and B with 2.0 equilvalents Zn^{2+}.

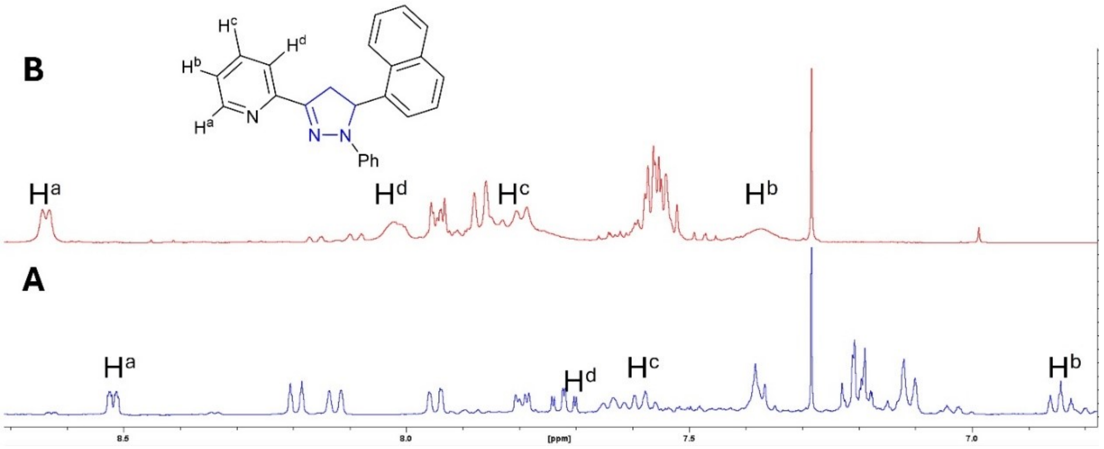

Partial ^{1}H NMR spectra for **2** only A (20 µM, $CDCl_3$) and B with 2.0 equilvalents Zn^{2+}.

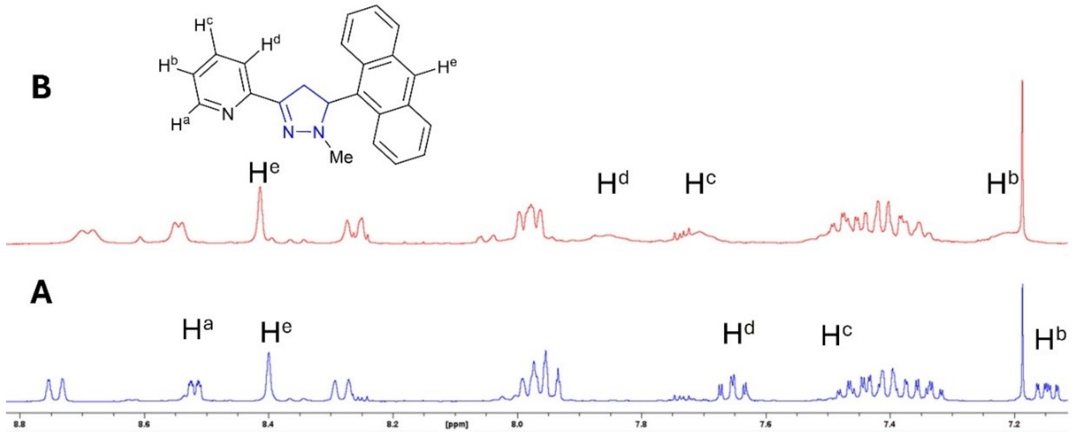

Partial ^{1}H NMR spectra for **3** only A (20 µM, $CDCl_3$) and B with 2.0 equilvalents Zn^{2+}.

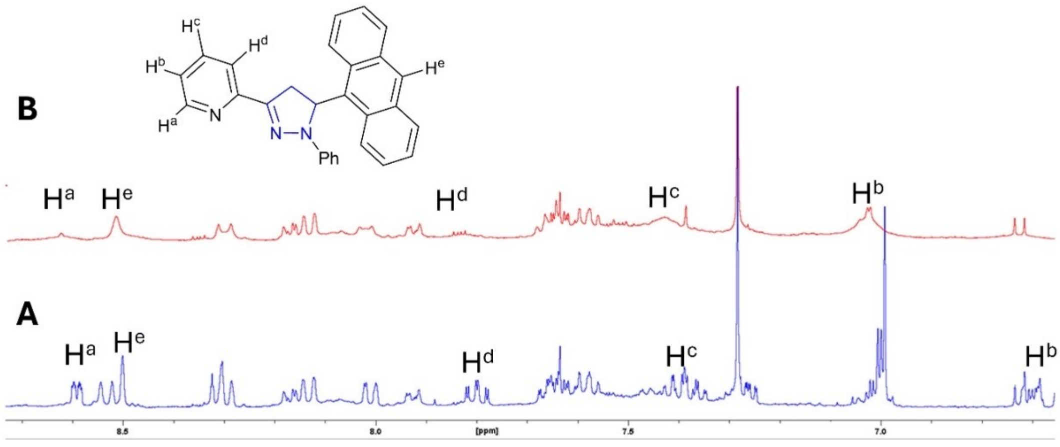

Partial ^{1}H NMR spectra for **4** only A (20 μM, $CDCl_3$) and B with 2.0 equilvalents Zn^{2+}.

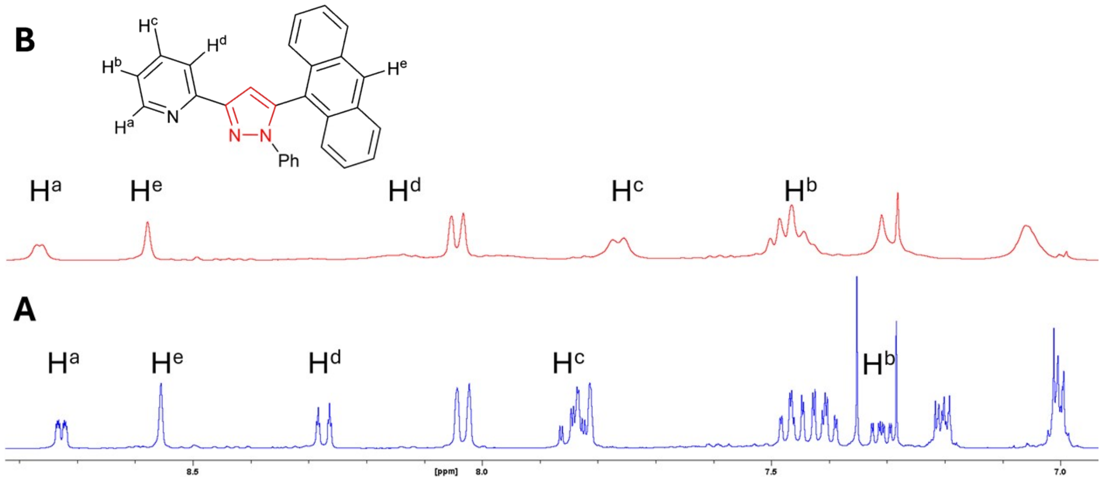

Partial ^{1}H NMR spectra for 7 only A (15 μM, $CDCl_3$) and B with 2.0 equilvalents Zn^{2}.

UV/Vis Spectroscopy (S4)

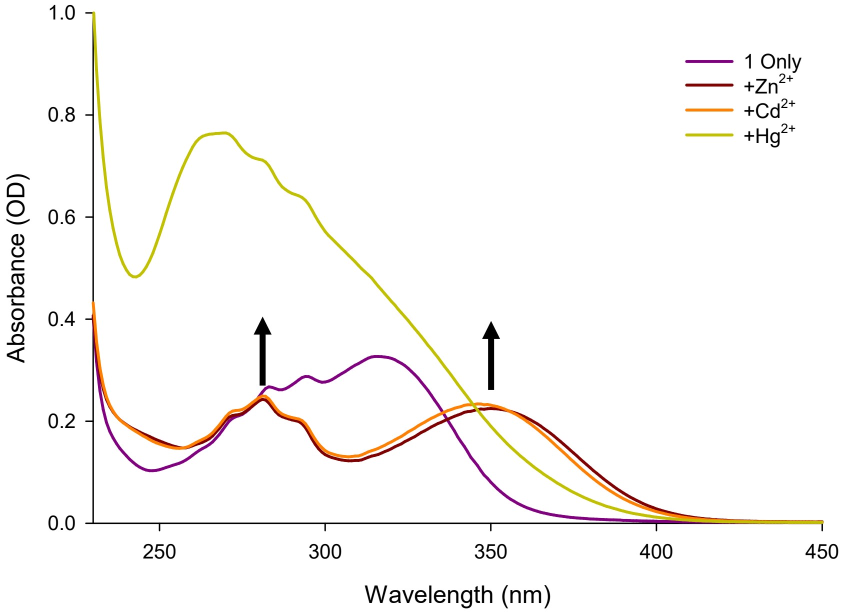

UV/Vis study of **1** only (20 µM, MeCN) and with 5 equivalents Zn^{2+}, Cd^{2+} and $+Hg^{2+}$. Arrow indicates change in bands.

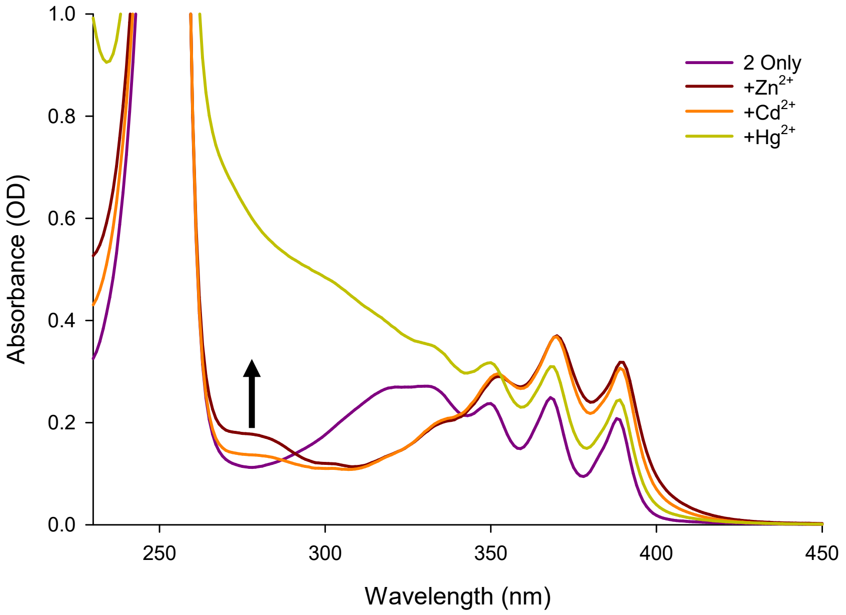

UV/Vis study of **2** only (20 µM, MeCN) and with 5 equivalents Zn^{2+}, Cd^{2+} and $+Hg^{2+}$. Arrow indicates change in bands.

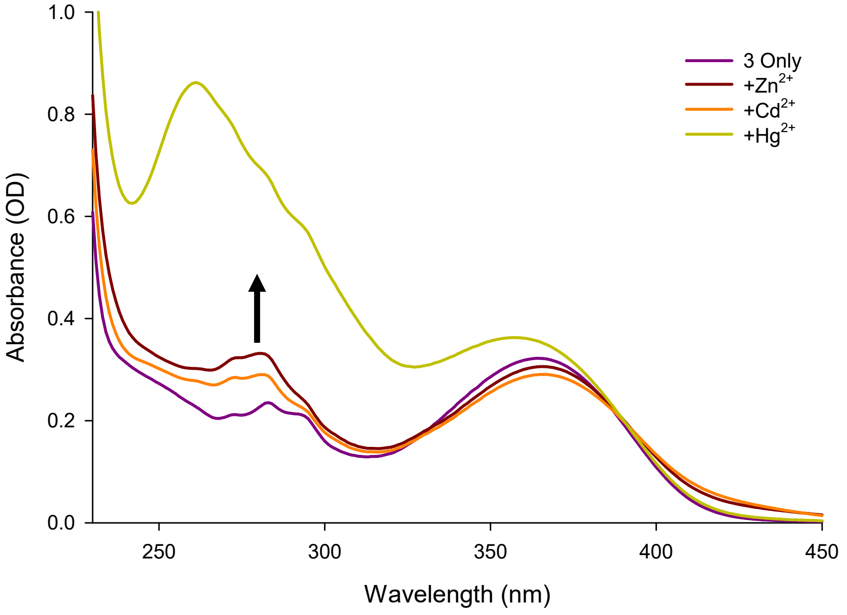

UV/Vis study of **3** only (20 µM, MeCN) and with 5 equivalents Zn^{2+}, Cd^{2+} and $+Hg^{2+}$. Arrow indicates change in bands.

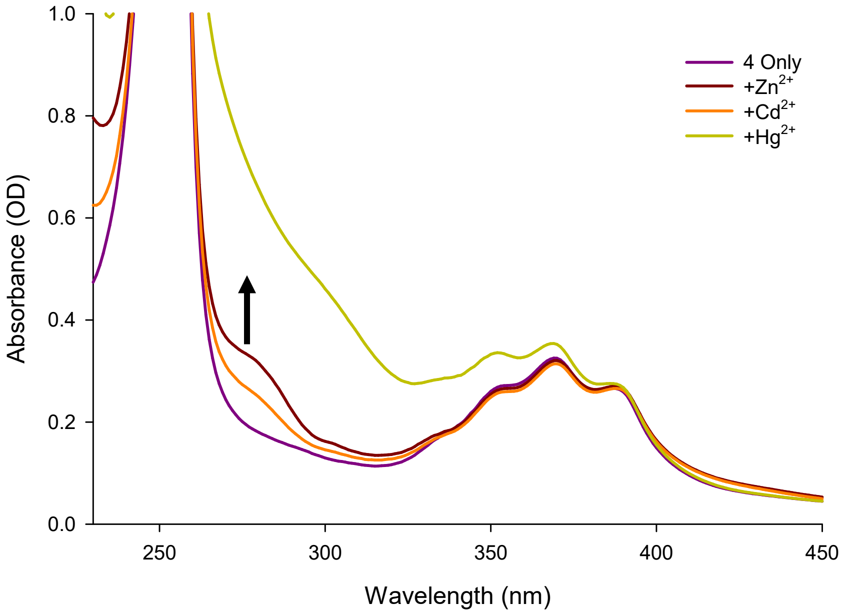

UV/Vis study of **4** only (20 µM, MeCN) and with 5 equivalents Zn^{2+}, Cd^{2+} and $+Hg^{2+}$. Arrow indicates change in bands.

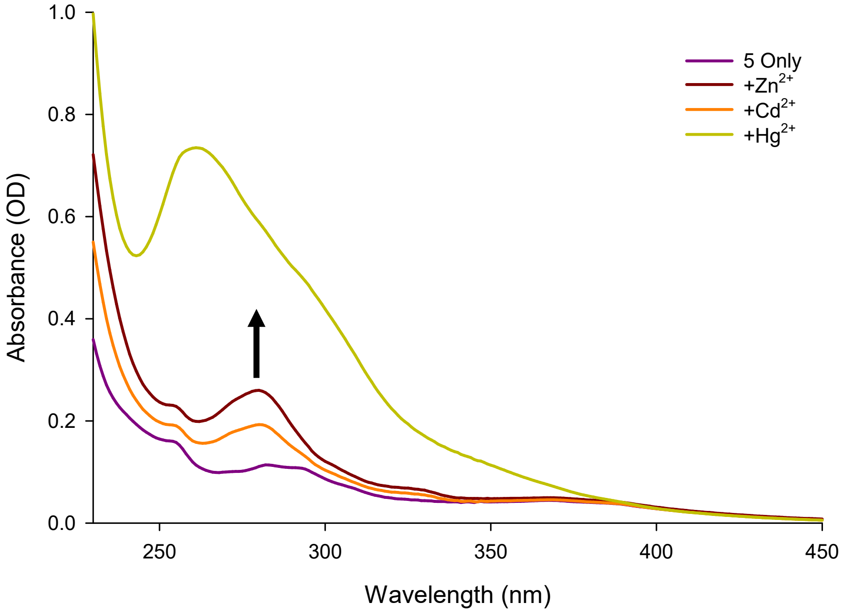

UV/Vis study of **5** only (20 µM, MeCN) and with 5 equivalents Zn^{2+}, Cd^{2+} and +Hg^{2+}. Arrow indicates change in bands.

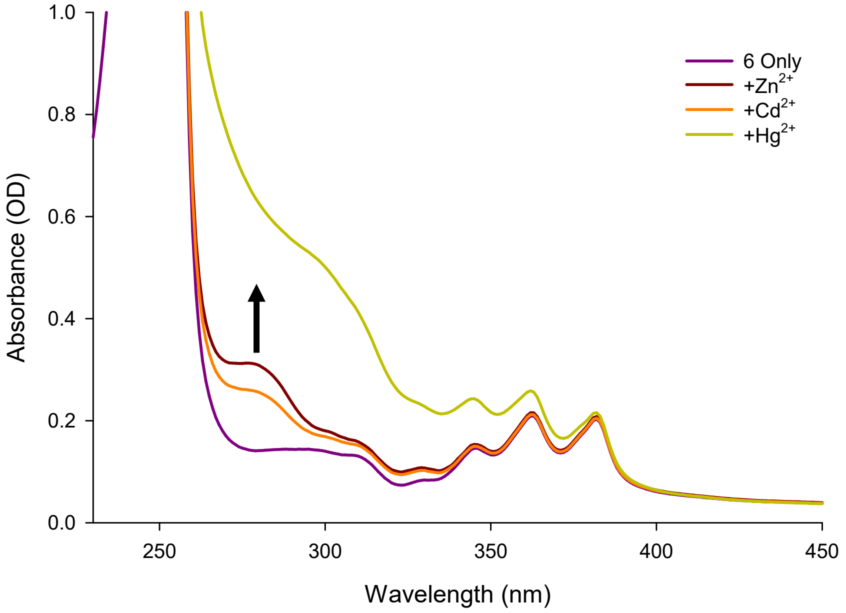

UV/Vis study of **6** only (20 µM, MeCN) and with 5 equivalents Zn^{2+}, Cd^{2+} and +Hg^{2+}. Arrow indicates change in bands.

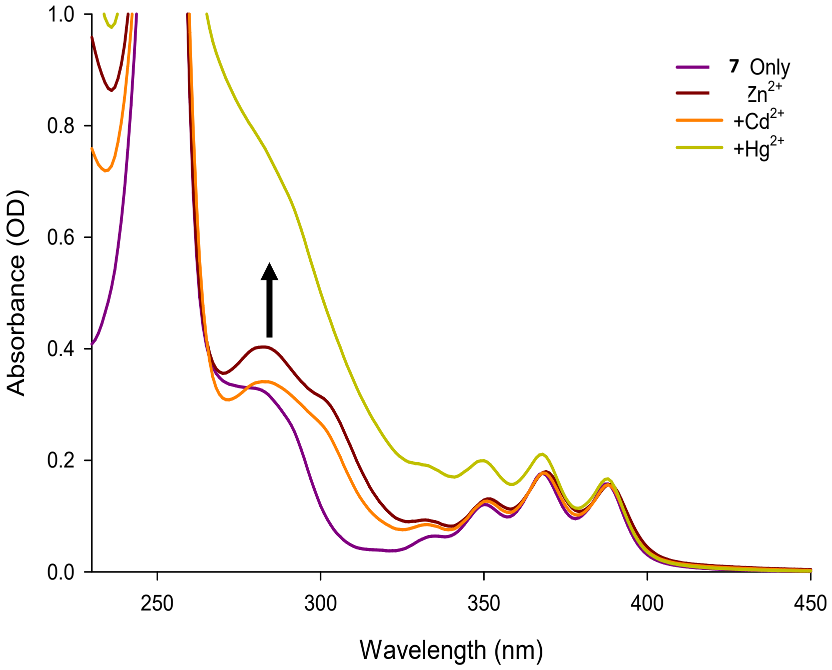

UV/Vis study of **7** only (20 µM, MeCN) and with 5 equivalents Zn^{2+}, Cd^{2+} and +Hg^{2+}. Arrow indicates change in bands.

Aqueous study of pyrazoline **1** in 7:3 MeCN:H_2O solution on addition of Cd^{2+} (0-10 equivalent), λ_{ex} 280 nm (S5)

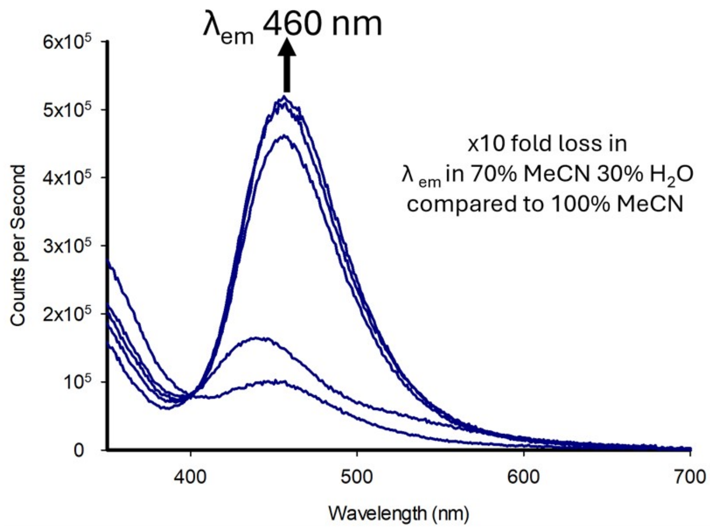

Aqueous study of sensor **2** (20 μM, MeCN) with 5.0 equivalents indicated metal in 7:3 MeCN:H_2O solution at λ_{ex} 280 nm, cps is counts per second

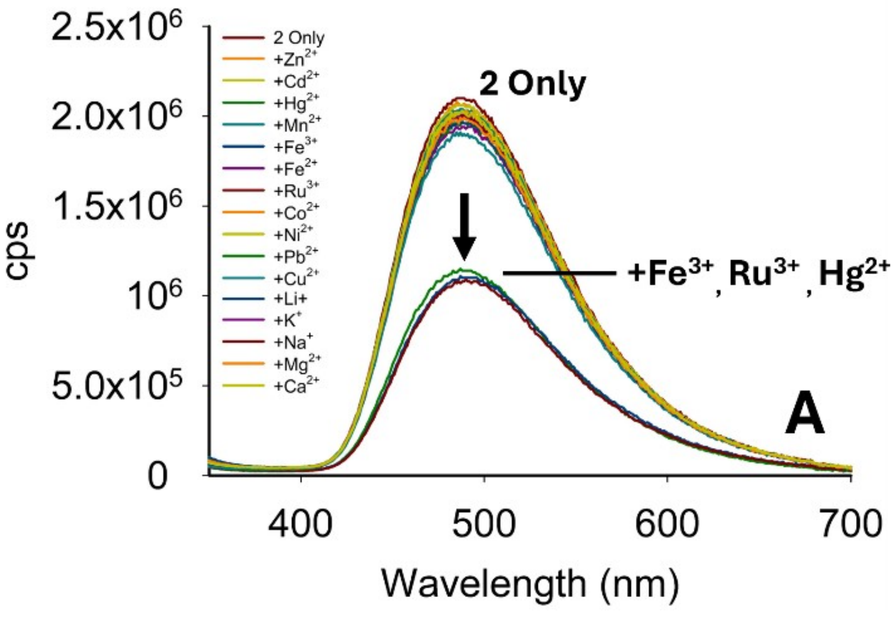

Aqueous study of sensor **7** (20 µM, MeCN) with 5.0 equivalents indicated metal in 7:3 MeCN:H_2O solution at λ_{ex} 250 nm, cps is counts per second

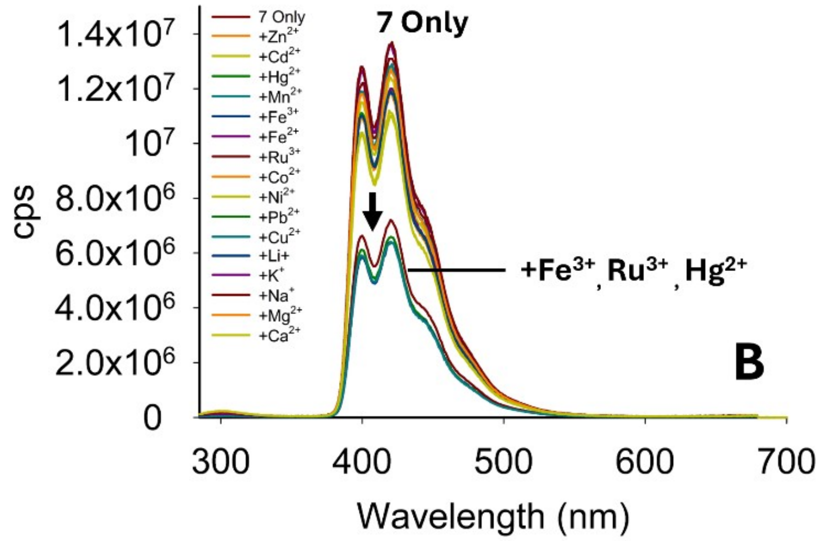

Real-world example of pyrazoline **2** in tap water panel A and mineral water panel B, solution was 7:3 MeCN:H_2O, **2** concentration 20 µM, λ_{ex} 280 nm (S6)

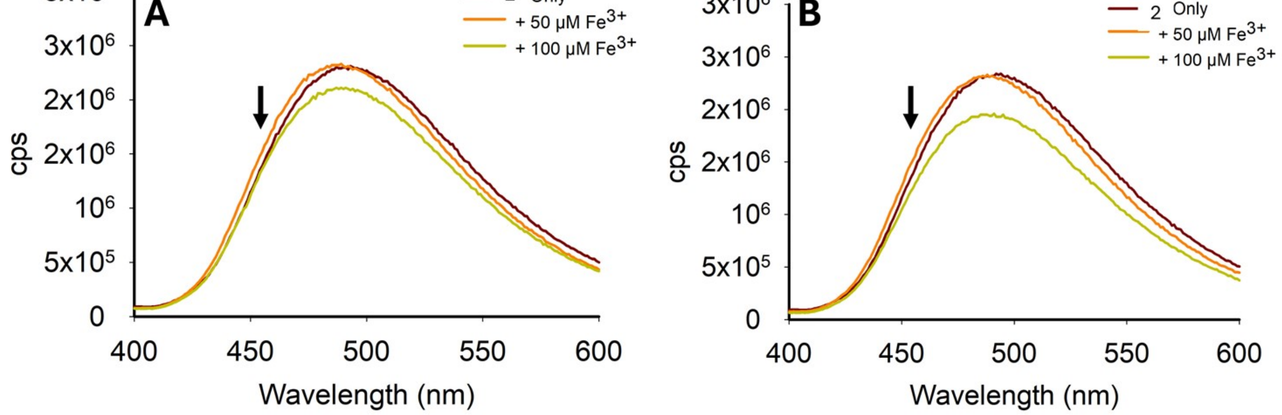

Real-world example of pyrazoline **2** in pond water panel A and river water panel B, solution was 7:3 MeCN:H_2O, **2** concentration 20 μM, λ_{ex} 280 nm (S7)

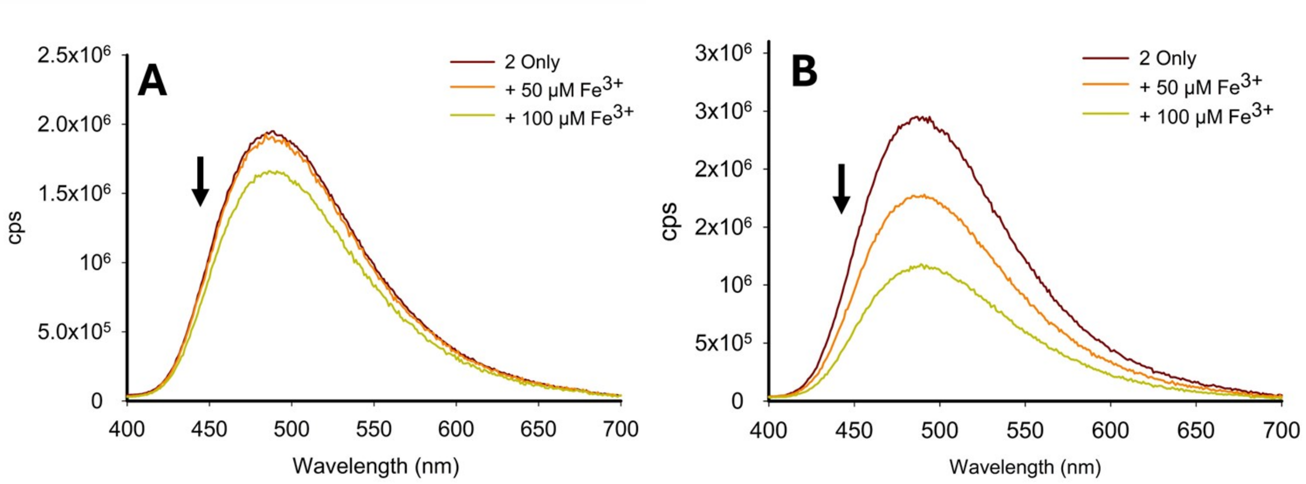

Limit of Detection (LoD) Studies (S8)

The method reported by Lee *et al* was used to calculate limit of detection (LoD) for **2** (λ_{ex} 280 nm) and **7** (λ_{ex} 250 nm) in 7:3 MeCN:H_2O with the average from two replicates used.

$$LoD = 3\sigma_{bi}/m$$

σ_{bi} = standard deviation of sensor only (n=10)

m = gradient of the slope, note the inverse sign was used as these are "turn off" sensors not "turn on"

B. P. Joshi, J. Park, W. I. Lee and K.-H. Lee, Talanta, 2009, 78, 903.

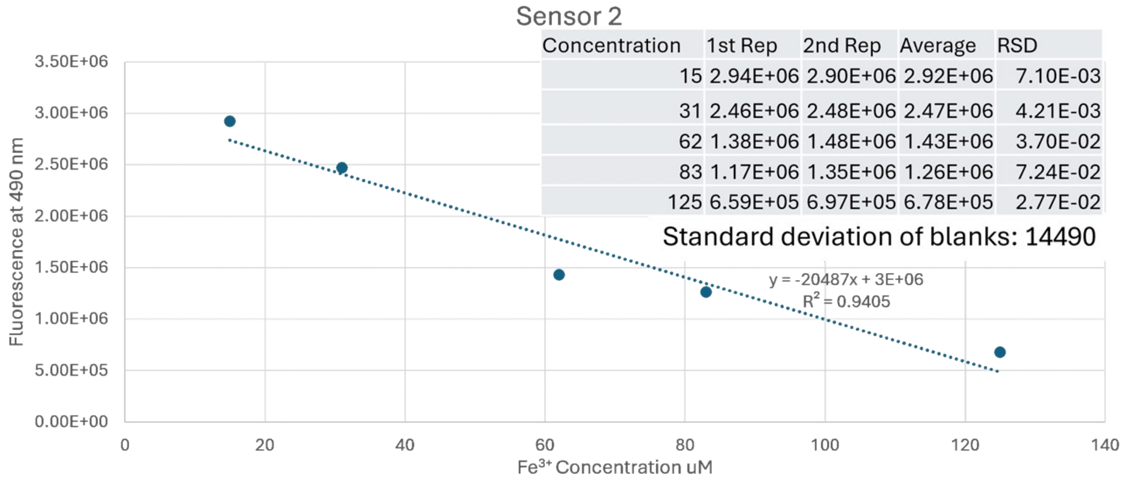

Concentration	1st Rep	2nd Rep	Average	RSD
15	2.94E+06	2.90E+06	2.92E+06	7.10E-03
31	2.46E+06	2.48E+06	2.47E+06	4.21E-03
62	1.38E+06	1.48E+06	1.43E+06	3.70E-02
83	1.17E+06	1.35E+06	1.26E+06	7.24E-02
125	6.59E+05	6.97E+05	6.78E+05	2.77E-02

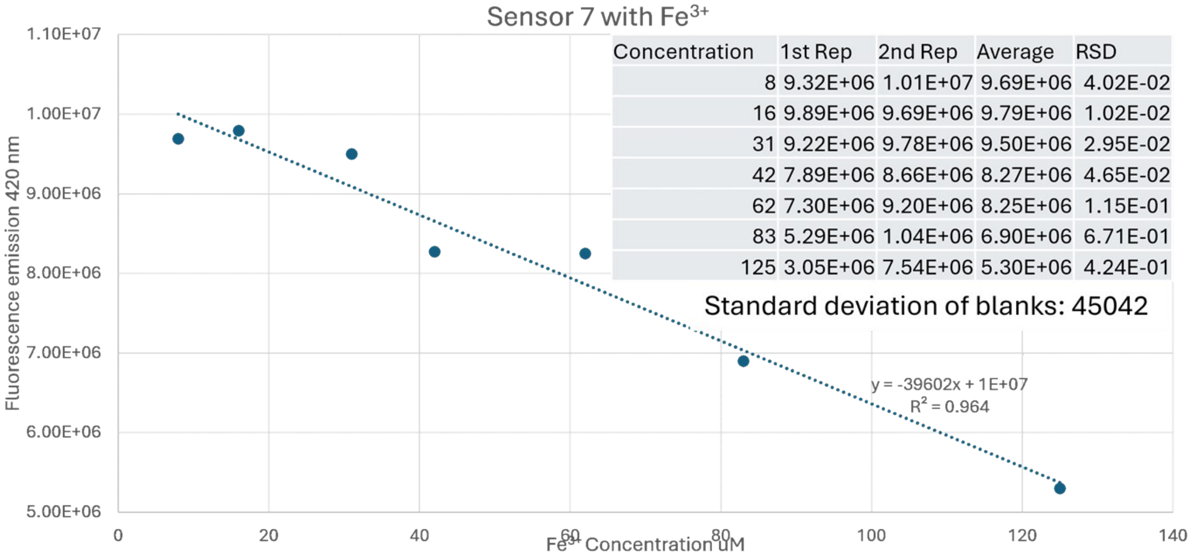

Concentration	1st Rep	2nd Rep	Average	RSD
8	9.32E+06	1.01E+07	9.69E+06	4.02E-02
16	9.89E+06	9.69E+06	9.79E+06	1.02E-02
31	9.22E+06	9.78E+06	9.50E+06	2.95E-02
42	7.89E+06	8.66E+06	8.27E+06	4.65E-02
62	7.30E+06	9.20E+06	8.25E+06	1.15E-01
83	5.29E+06	1.04E+06	6.90E+06	6.71E-01
125	3.05E+06	7.54E+06	5.30E+06	4.24E-01

Analyte	Structure	Detection	Mode	LoD	Solvent	Reference
Fe^{3+}	Pyrazoline	Fluorescence	"Turn off"	0.401 μM	$THF:H_2O$ = 9:1	*J. Fluoresc.*, 2024, **34**, 159
Fe^{3+}	Pyrazole	Fluorescence	"Turn off"	1.86 μM	$EtOH:H_2O$= 7:3	*Org. Biomol. Chem.*, 2023, **21**, 4482
Fe^{3+}	Pyrazole	Fluorescence	"Turn off"	21 nM	$DMSO:H_2O$= 9:1	*J. Photochem. Photobiol., A*, 2023, **437**, 114470
Fe^{3+}	Pyrazole	Fluorescence	"Turn off"	45 nM	$DMSO:H_2O$= 9:1	*New J. Chem.*, 2023, **47**, 751
Fe^{3+}	Pyrazole	Fluorescence	"Turn off"	21 nM	$DMSO:H_2O$= 9:1	*J. Photochem. Photobiol., A*, 2023, **437**, 114470
Fe^{3+}	Pyrazoline	Fluorescence	"Turn off"	0.12 μM	$MeOH:H_2O$ = 1:9	*J. Fluoresc.*, 2022, **32**, 2319
Fe^{3+}	Pyrazole	Fluorescence	"Turn on"	1.73 μM	$MeCN:H_2O$ = 7:3	*Spectrochim. Acta, Part A*, 2020, **230**, 117993
Fe^{3+}	Quinazoline	Fluorescence	"Turn on"	3.5 μM	$MeCN:H_2O$= 2:8	*Analyst*, 2012, **137**, 3335
Fe^{3+}	Rhodamine–naphthalic anhydride	Fluorescence	"Turn on"	2.90 μM	$MeOH:H_2O$= 6:4	*Dalton Trans.*, 2015, **44**, 11805

Selection of recently reported Fe^{3+} LoD in a range of solvents

Quantum Yield calculated using the absolute method and an integrating sphere (S9)

Sensor	Solvent		Quantum Yield ϕ
**S2**	MeCN	**S2** only	0.74
**S2**	MeCN	**S2** + Fe^{3+}	<0.01
**S2**	$MeCN:H_2O$ 7:3	**S2** only	0.83
**S2**	$MeCN:H_2O$ 7:3	**S2** + Fe^{3+}	0.07
**S7**	MeCN	**S7** only	0.33
**S7**	MeCN	**S7** + Fe^{3+}	<0.01
**S7**	$MeCN:H_2O$ 7:3	**S7** only	0.43
**S7**	$MeCN:H_2O$ 7:3	**S7** + Fe^{3+}	0.18

Recovery study for 2 and 7 (S10)

Sensor	Solvent	cps	% Fe^{3+} Recovery
Sensor **2** + 50 µM Fe^{3+}	Mineral Water	2.309 x10^6	64%
Sensor **2** + 100 µM Fe^{3+}	Mineral Water	1.949 x10^6	40%
Sensor **2** + 50 µM Fe^{3+}	Tap Water	2.314 x10^6	64%
Sensor **2** + 100 µM Fe^{3+}	Tap Water	2.080 x10^6	38%
Sensor **2** + 50 µM Fe^{3+}	Pond Water	1.870 x10^6	80%
Sensor **2** + 100 µM Fe^{3+}	Pond Water	1.640 x 10^6	48%
Sensor **2** + 50 µM Fe^{3+}	River Water	1.760 x 10^6	68%
Sensor **2** + 100 µM Fe^{3+}	River Water	1.170 x 10^6	48%

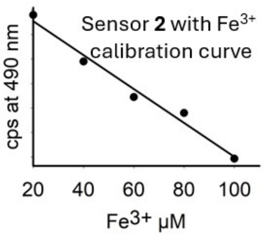

Sensor	Solvent	cps	% Fe^{3+} Recovery
Sensor **7**+ 50 µM Fe^{3+}	Mineral Water	9.435 x10^6	92%
Sensor **7** + 100 µM Fe^{3+}	Mineral Water	7.859 x10^6	66%
Sensor **7** + 50 µM Fe^{3+}	Tap Water	9.561 x10^6	91%
Sensor **7** + 100 µM Fe^{3+}	Tap Water	7.956 x10^6	65%
Sensor **7** + 50 µM Fe^{3+}	Pond Water	6.920 x 10^6	125%
Sensor **7** + 100 µM Fe^{3+}	Pond Water	5.750 x10^6	90%
Sensor **7** + 50 µM Fe^{3+}	River Water	5.980 x 10^6	145%
Sensor **2** + 100 µM Fe^{3+}	River Water	5.360 x 10^6	97%

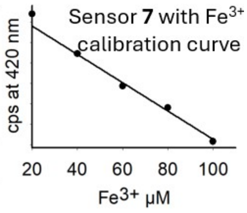

Job Plot for 7 (S11)

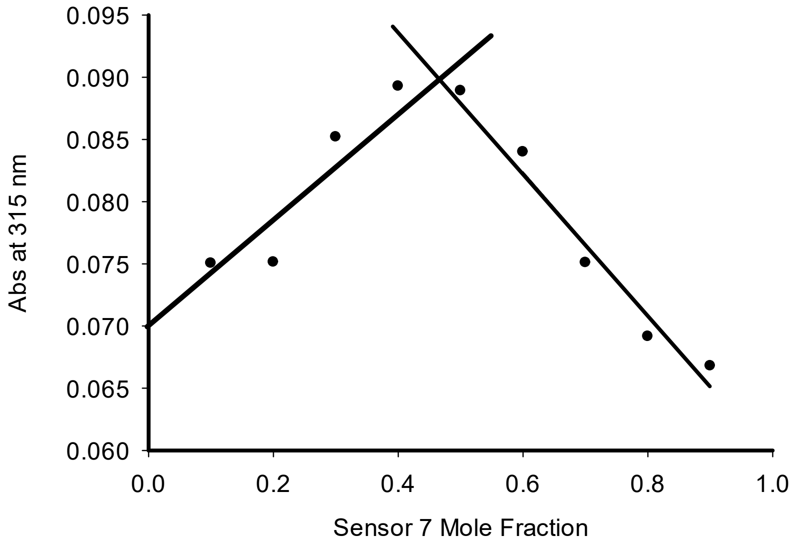

Job plot for **7** with Fe^{3+}, [Fe^{3+}] + [**7**] = 100 µM, MeCN
